# *WAS* Promoter-Driven Lentiviral Vectors Mimic Closely the Lopsided WASP Expression during Megakaryocytic Differentiation

**DOI:** 10.1016/j.omtm.2020.09.006

**Published:** 2020-09-16

**Authors:** Pilar Muñoz, María Tristán-Manzano, Almudena Sánchez-Gilabert, Giorgia Santilli, Anne Galy, Adrian J. Thrasher, Francisco Martin

**Affiliations:** 1 Genomic Medicine Department, GENYO, Centre for Genomics and Oncological Research, Pfizer-University of Granada-Andalusian Regional Government, Parque Tecnológico Ciencias de la Salud (PTS), Avenida de la Ilustracion 114, 18016 Granada, Spain; 2 University College London (UCL) Great Ormond Street Institute of Child Health (ICH), 30 Guilford Street, WC1N 1EH London, UK; 3 Genethon, 91000 Evry, France; 4 Université Paris-Saclay, Univ Evry, Inserm, Genethon, Integrare research unit UMR_S951, 91000 Evry, France

**Keywords:** Wiskott-Aldrich syndrome, alternative *WAS* promoter, hematopoietic stem and progenitor cells, HSPCs, WASKO cellular models, megakaryocytic differentiation, lentiviral vectors, phenotypic rescue, WAS patients, physiollogical expression, gene therapy

## Abstract

Transplant of gene-modified autologous hematopoietic progenitors cells has emerged as a new therapeutic approach for Wiskott-Aldrich syndrome (WAS), a primary immunodeficiency with microthrombocytopenia and abnormal lymphoid and myeloid functions. Despite the clinical benefits obtained in ongoing clinical trials, platelet restoration is suboptimal. The incomplete restoration of platelets in these patients can be explained either by a low number of corrected cells or by insufficient or inadequate WASP expression during megakaryocyte differentiation and/or in platelets. We therefore used *in vitro* models to study the endogenous WASP expression pattern during megakaryocytic differentiation and compared it with the expression profiles achieved by different therapeutic lentiviral vectors (LVs) driving *WAS* cDNA through different regions of the *WAS* promoter. Our data showed that all *WAS* promoter-driven LVs mimic very closely the endogenous *WAS* expression kinetic during megakaryocytic differentiation. However, LVs harboring the full-length (1.6-kb) *WAS*-proximal promoter (WW1.6) or a combination of the *WAS* alternative and proximal promoters (named AW) had the best behavior. Finally, all *WAS*-driven LVs restored the WAS knockout (WASKO) mice phenotype and functional defects of hematopoietic stem and progenitor cells (HSPCs) from a WAS patient with similar efficiency. In summary, our data back up the use of WW1.6 and AW LVs as physiological gene transfer tools for WAS therapy.

## Graphical Abstract

**Figure:**
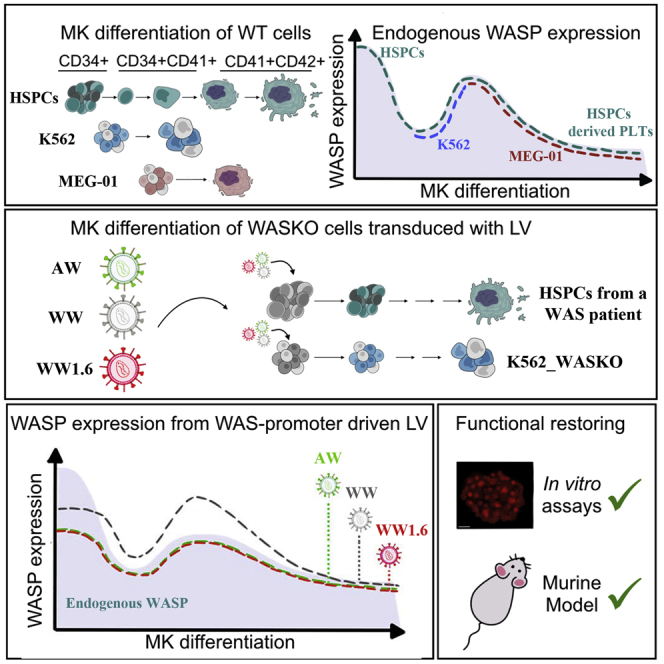

## Teaser

In this study, Martin et al. have uncovered the lopsided WASP expression kinetic during human megakaryocyte differentiation, showing that *WAS* promoter-driven LVs follow closely this expression pattern. Based on these analyses, the authors propose a new clinical trial with the AW LVs incorporating additional regulatory sequences from the alternative WAS promoter.

## Introduction

Wiskott-Aldrich syndrome (WAS) is an X-linked rare primary immunodeficiency (incidence of 1 in 10^5^ to 1 in 10^6^ cases per live birth) (OMIM: 301000) and makes up approximately 3% of all primary immunodeficiency disorders. It is diagnosed early in life, and many patients with severe WAS do not survive past 10 years of age without definitive treatment.^1^ The classical WAS phenotype is characterized by eczema, immunodeficiency, microthrombocytopenia, autoimmunity, and malignancies.^2^ WAS is caused by mutations in the *WAS* gene^3^ (gene map locus Xp11.23-p11.22) that are expressed exclusively in hematopoietic cells and play important roles in signaling and actin cytoskeleton reorganization (reviewed in Blundell et al.^4^). Therefore, most hematopoietic cells are affected to different degrees, causing the phenotypic abnormalities observed in patients with WAS. Of all functional defects, increased bleeding due to low platelet (PLT) counts is the major challenge, with up to 30% of WAS patients suffering life-threatening bleeding episodes.^5^

The only curative treatments for WAS patients are allogeneic hematopoietic stem and progenitor cell (HSPC) transplantation^6^ and autologous gene-modified HSPCs.^7^ Although HSPC transplantation is the standard treatment procedure and is usually curative, the use of human leukocyte antigen (HLA)-matched HSPCs is associated with acute morbidity and a high incidence of long-term complications, although overall survival rates are continually improving.^8^, ^9^, ^10^ On the contrary, lentiviral-based gene therapy (GT) clinical trials observed limited toxicity and similar survival rates,^7^^,^^11^, ^12^, ^13^, ^14^ making this treatment a rational alternative therapeutic option. In these trials, autologous CD34^+^ cells were genetically modified using a self-inactivating (SIN) lentiviral vector (LV) expressing WASP under a 1.6-kb fragment of the proximal promoter of the *WAS* gene.^15^ A general consensus that arose from these clinical trials is that, although immune deficiency was corrected, complete remission of microthrombocytopenia was more difficult to achieve.^16^ Of note, HSPC transplantation is more effective than GT in this aspect and is therefore a drawback to overcome in new WAS GT products.^16^

The reasons behind the low PLT recovery achieved with GT in most WAS patients are unclear. It could be due to the absence of the selective advantage of WASP-expressing PLTs,^17^ to the suboptimal WASP expression in megakaryocytes (MKs), MK progenitors, and/or PLTs,^14^ or to a combination of both factors. In this direction, our hypothesis is that a physiological expression of WASP would improve GT outcomes by preventing potential side effects of underexpression or overexpression of WASP along MK differentiation. The exact functions of WASP in PLTs remains largely unknown, but there is strong evidence suggesting that it plays a critical role regulating MK differentiation and PLT formation by inhibiting these processes in the absence of the appropriated signals.^18^, ^19^, ^20^ This could sound contradictory with the thrombocytopenia found in WAS patients, but it is actually a potential explanation for it. Indeed, PLTs produced in the absence of WASP have not been developed properly and have a reduced size, abnormal ultrastructure, and surface markers that lead to their elimination in the spleen and other tissues. Therefore, in order to generate normal PLT levels in WAS patients, we should not only engraft with the appropriate levels of WASP-expressing HSPCSs, but also mimic very closely the physiological expression of WASP during MK development.

Different groups, including ours, have developed physiologically regulated LVs for the treatment of WAS using different fragments of the *WAS-*proximal promoter to drive the expression of *WAS* cDNA.^15^^,^^21^ These studies have shown hematopoietic-specific expression of the different *WAS* promoter-driven LVs that efficiently restored WASP defects in animal models.^22^^,^^23^ Later studies also showed improved safety of these LVs by avoiding WASP expression in non-hematopoietic cells^24^ and reducing genotoxicity.^25^ These works led to the approval of the clinical trials mentioned above using the 1.6-kb *WAS*-proximal promoter-driven LVs.^15^ However, as referred to previously, despite the good clinical results, these trials showed suboptimal WASP expression in PLTs and suboptimal recovery of microthrombocytopenia in most patients. In an attempt to improve the behavior of *WAS*-proximal promoter-driven LVs, our group generated AWE LVs^26^ that harbored regulatory fragments from the two *WAS* promoters described in the literature, i.e., the proximal promoter^27^ and the alternative promoter.^28^ The proximal promoter starts immediately upstream of the transcription start site (GenBank: AC115618.3 sequence [Seq] 120189–121855), and the alternative promoter is located 6 kb upstream (GenBank: AC115618.3 Seq 114403–115000). The AWE LVs showed improved enhanced GFP (EGFP) expression in myeloid, megakaryocytic, and B cell lineages compared to *WAS*-proximal promoter-driven LVs.^26^ However, since the *WAS* cDNA contains regulatory sequences that affect its expression pattern,^29^ it is fundamental to study the behavior of the backbone of AW LVs expressing *WAS* cDNA in order to determine whether they achieve truly physiological expression. In this work we use *in vitro* models to study whether *WAS* promoter-driven LVs harboring sequences from the alternative promoter could improve their therapeutic potential by mimicking more closely the WASP expression pattern during megakaryocytic differentiation. Although WASP expression levels are well documented in all mature blood cells,^30^^,^^31^ the precise WASP expression profile in HSPCs along MK differentiation and PLT generation is mostly unknown. We therefore analyzed first the WASP expression kinetic during MK differentiation and then compared it with the expression pattern of the different LVs driving the transgene through different regions of the *WAS* promoter, including the LVs used in ongoing clinical trials for WAS. Our data showed that all *WAS* promoter-driven LVs mimic very closely the WASP endogenous expression kinetic during MK differentiation. We also studied potential therapeutic improvements of the AW LVs compared with the WW 1.6-kb (WW1.6) LVs, but could not find any significant differences.

## Results

### WASP Endogenous Expression Decreases during *In Vitro* Megakaryopoiesis and Thrombopoiesis

**Figure 1:**
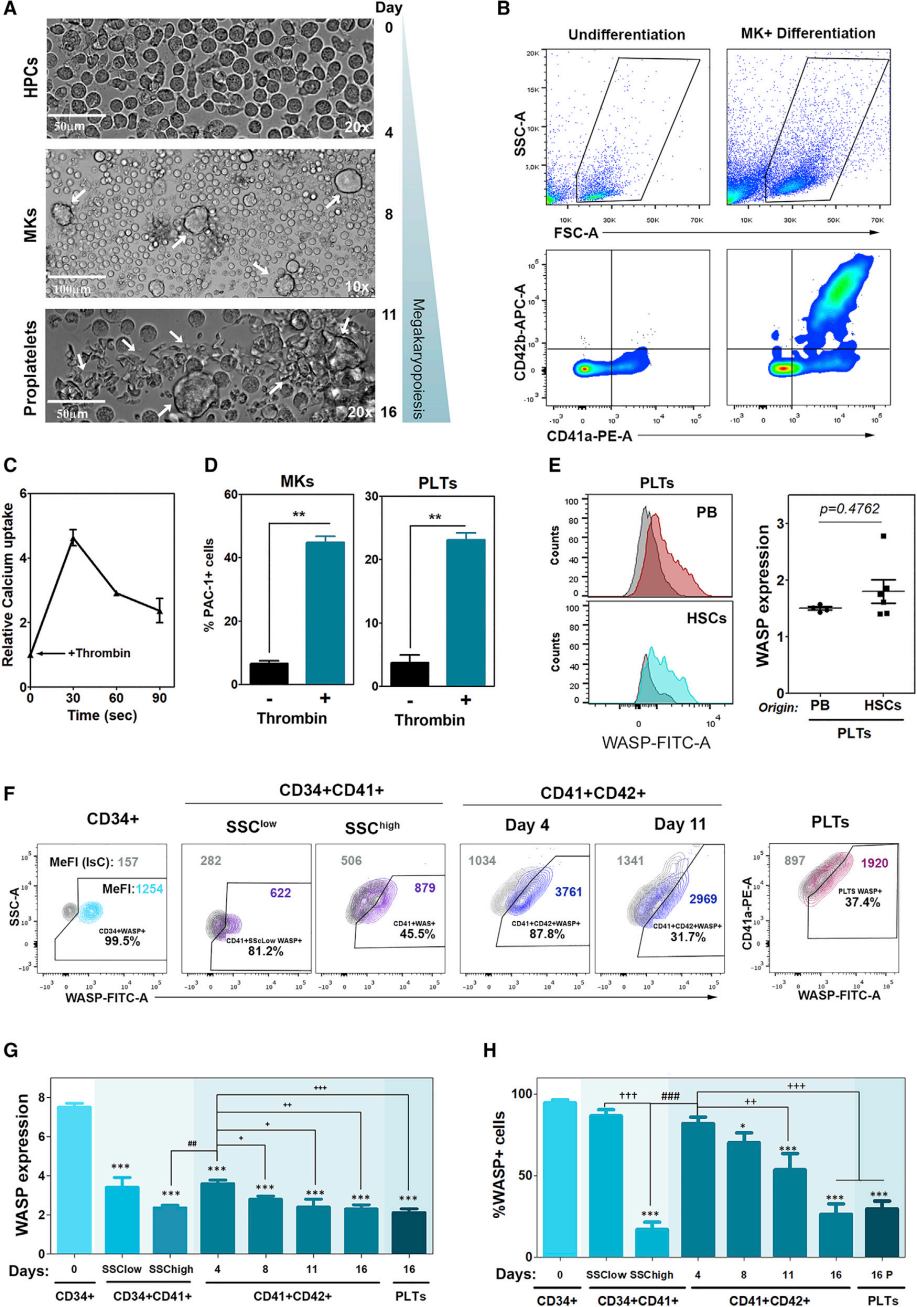
WASP Endogenous Expression Decreases during *In Vitro* Megakaryopoiesis and Thrombopoiesis (A) Representative bright-field images of hHSPCs before (top panel, 0–4 days) and after 4–8 days (middle panel, MKs are indicated with white arrows) or 11–16 days (bottom panel, MKs releasing proplatelets, white arrows) of MK differentiation with TPO and SCF without iROCK as indicated in Materials and Methods. (B) Representative plots showing changes in morphology (FSC^high^SSC^high^) and appearance of megakaryocytic markers (CD41a and CD42b) of HSPCs cultured with the MK differentiation media. (C) Mature MKs (FSC^high^SSC^high^CD42^+^ cells) respond to thrombin by increasing intracellular calcium uptake (n= 3). (D) Expression of PAC-1 activation marker in response to thrombin in obtained MKs (FSC^high^SSC^high^CD42^+^ cells) and PLTs (FSC^low^SSC^low^CD42^+^ cells) (n = 3). (E) Representative histograms showing WASP expression levels of PLTs (FSC^low^SSC^low^CD41^+^CD42^+^) obtained from peripheral blood (PB, top left) and HSPCs (bottom left). WASP expression (related to isotype control [IsC]) (right) is represented as mean ± SEM (non-parametric Mann-Whitney test, two-tailed). (F) Representative dot plots of endogenous WASP expression kinetics upon MK differentiation *in vitro*. Grey populations in each plot display the IsC staining and colored populations display the WASP staining (n = 7). (G) Analysis of WASP expression, depicted as MeFI of WASP^+^ population/MeFI of IsC, in the different populations during MK differentiation. CD34^+^ indicates HSPCs at day 0 (CD34^+^CD41^−^CD42^−^), MK progenitors (CD34^+^CD41^+^SSC^low^SSC^high^) at days 4–8, mature MKs (CD34^+^CD41^+^CD42^+^) during 4–16 days of differentiation, and PLTs (CD41^+^CD42^+^FSC^low^SSC^low^) obtained at day 16. (H**)** Percentage of WASP^+^ cells in the above-described populations. Represented data are mean ± SEM. ∗p < 0.05, ∗∗p < 0.01, ∗∗∗p < 0.001 (non-parametric Mann-Whitney test, two-tailed, compared to CD34^+^ expression levels) (n = 7).

**Figure 2:**
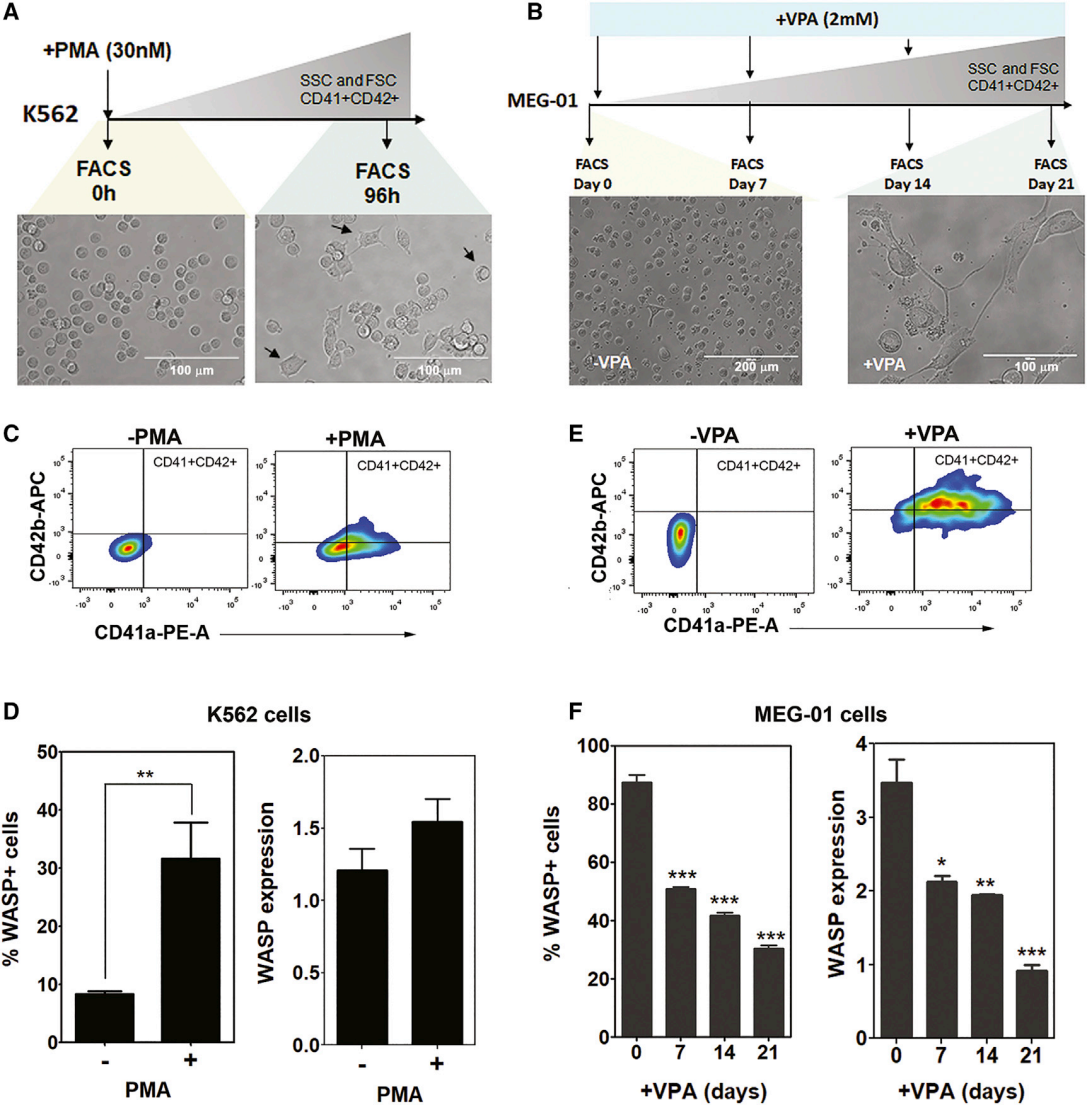
Immortalized “MK-like” Cell Lines Are Relevant Models to Study Wasp Expression during MK Differentiation (A) Scheme of megakaryocytic differentiation protocol of K562 cells (top) and associated morphological changes after 96 h of PMA incubation. (B) Diagram of MK differentiation protocol of MEG-01 cells (top) and morphological changes after VPA addition. (C) Representative FACS plots of CD41a and CD42b megakaryocytic markers of K562 cells in the absence or presence of PMA. (D) Percentage of WASP^+^ K562 cells and WASP expression without PMA treatment, analyzed in the total population (n = 4). (E) Phenotypic changes of MEG-01 cells after VPA treatment expressing CD41a and CD42b markers. (F) Percentage of WASP^+^ MEG-01 cells and WASP expression at 0, 7, 14, and 21 days of VPA-megakaryocytic differentiation (n = 3). Represented data are means ± SEM, non-parametric Mann-Whitney test, two-tailed. ∗∗p < 0.01, ∗∗∗p < 0.001.

Since we wanted to mimic WASP endogenous expression during MK differentiation, we first established primary (Figure 1) and immortalized (Figure 2) cellular models to study WASP expression patterns through the MK differentiation process. MK progenitors (CD34^+^CD41^+^), MKs (CD34^−^CD41^+^CD42^+^), and PLTs (CD41^+^CD42^+^FSC^low^SSC^low^) were routinely obtained from HSPCs (CD34^+^CD41^−^CD42^−^) using the protocol depicted in Figure S1A, where human CD34^+^ cells were incubated with stem cell factor (SCF), thrombopoietin (TPO), and ROCK inhibitor as described in Materials and Methods during 16 days. We could observe large cells that correlate with immature MKs (Figure 1A, center) that finally extend proplatelets (Figure 1A, bottom) and are also associated with the phenotypical acquisition of CD41a and CD42b megakaryocytic markers (Figure 1B). In addition, PLTs derived from HSPCs were functional in response to thrombin (Figures 1C and 1D) and expressed similar WASP protein levels as did those PLTs isolated from peripheral blood of healthy donors (HDs) (Figure 1E), validating this cellular model to study MK differentiation. We then analyzed WASP expression levels (Figures 1F–1H) in MK progenitors, MKs, and PLTs at days 4, 8, 11, and 16 of MK differentiation following gate strategy analysis represented in Figure S1B. Briefly, we first selected three different gates according to forward scatter (FSC) and side scatter (SSC) (HPCs, MKs, and PLTs), and then we further gated (1) undifferentiated CD34^+^ cells; (2) MK early progenitors (CD34^+^CD41^+^ cells); (3) MK progenitors (SSC^high^CD34^+^CD41^+^); (4) MKs (CD41^+^CD42^+^SSC^high^); and (5) PLTs (CD41^+^CD42^+^SSC^low^) as populations of interest. WASP expression was calculated as the intensity ratio of WASP^+^ cells and the background intensity of the isotype control of the selected population (Figure S1B; see Materials and Methods). Our data showed a clear WASP downregulation as the HSPCs differentiate to MK progenitors (Figures 1F–1H; CD34^+^ versus CD34^+^CD41^+^, respectively), a slight WASP increment when MK progenitors (CD34^+^CD41^+^SSC^high^) differentiate into early MKs (CD34^+^CD41^+^CD42^+^, day 4), and a gradual downregulation as the MKs mature into PLTs (days 8, 11, and 16 and PLTs). The percentage of WASP^+^ cells showed the same described tendency but in a more dramatic manner.

We then validated these results in K562 and MEG-01 cell lines previously characterized to give rise to MK-like cells (Figures 2A and 2B) as determined by the acquisition of CD41a and CD42b megakaryocytic markers (Figures 2C and 2E). K562 cells differentiated with phorbol myristate acetate (PMA) and MEG-01 cells with valproic acid (VPA) constitute classical models to study megakaryocytic differentiation, which partially mimic some megakaryocytic characteristics in terms of phenotype, maturation, and function.^32^ Interestingly, WASP expression levels and percentage of positive cells were increased upon MK differentiation in K562 cells (Figure 2D), while MEG-01 cells showed a clear downregulation along differentiation time (Figure 2F). These data indicate that K562 cells behave similar to MK progenitors that differentiate into early MKs in the presence of PMA, while MEG-01 cells resemble early MKs that give rise to mature MKs and PLTs upon the addition of VPA.^32^ These two cell lines therefore provide additional cellular models to study LV behavior during MK differentiation.

### *WAS* Promoter-Driven LVs Mimic WASP Expression Kinetics during MK Differentiation and Restored PLT Actin Nodule Formation

**Figure 3:**
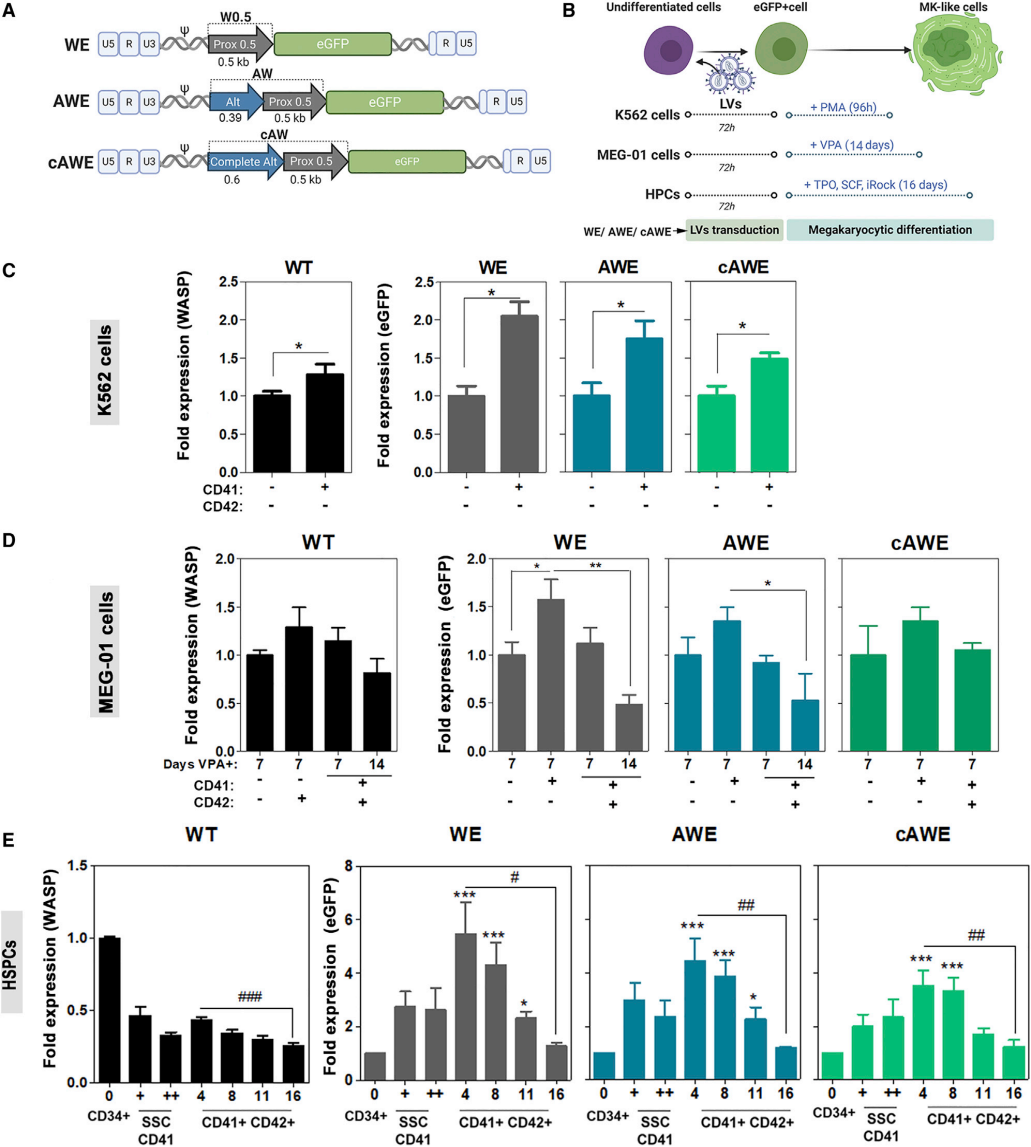
EGFP-LV Expression Driven by WAS Promoter Mimic Endogenous WAS Protein Expression Kinetics during Megakaryocytic Differentiation (A) Schematic representation of the second-generation LVs used in this study expressing enhanced GFP (EGFP). WE^21^ harbors the 500-bp core of the *WAS* proximal promoter (GenBank: AC115618.3 Seq 121356–121851), while AWE^26^ (GenBank: AC115618.3: Seq 114605–114991 linker of 17bp/ Seq 121356–121851) and cAWE (GenBank: AC115618.3: Seq 114403–115000 linker of 17 bp / Seq 121356–121851) contain additional 386-bp (Alt) and 597-bp (complete Alt) fragments of the *WAS* alternative promoter, respectively. (B) Experimental protocols for the comparison of WASP endogenous levels with EGFP expression given by the different LVs during MK differentiation in the different models. (C) Fold expression of endogenous WASP (black bars) and EGFP (colored bars) in K562 cells transduced with WE, AWE, and cAWE LVs (MOI of 1) are related to the expression levels of CD41^−^CD42^−^ cells in the PMA^+^ condition (non-parametric Mann-Whitney test, ∗p < 0.05) (n = 4). (D) WASP and EGFP fold expression at days 7 and 14 of MK differentiation of MEG-01 cells treated with VPA (non-parametric Mann-Whitney test, ∗p < 0.05, ∗∗p < 0.01) related to undifferentiated cells (n = 4). (E) WASP and EGFP fold expression in human CD34^+^, CD41^+^SSC^low^, CD41^+^SSC^high^, and CD41^+^CD42^+^ cells derived from HSPCs (endogenous WASP, black bars; EGFP, colored bars) at 4, 8, 11, and 16 days after *in vitro* MK differentiation (n = 10). Non-parametric Mann-Whitney test, two-tailed (^#^p < 0.05, ^##^p < 0.01, ^###^p < 0.001) and two-way ANOVA, Bonferroni post-test (∗∗∗p < 0.001) compared to wild-type (WT). All data are represented as mean ± SEM.

Once we established the three different human models of MK differentiation and their WASP expression kinetics, we proceeded to analyze the behavior of different second-generation LV backbones expressing the EGFP reporter gene through different fragments of the *WAS* promoter (Figures 3A and S2): the WE LV^21^ harbors the 500-bp core of the *WAS* proximal promoter, and the AWE^26^ and the cAWE (this work) contained additional sequences from the *WAS* alternative promoter. To analyze the physiological expression of these LVs during MK differentiation (Figure 3B), K562 cells (Figures 3C and S3A), MEG-01 cells (Figures 3D and S3B), and HSPCs (Figures 3E and S4) were transduced with the different LVs at a multiplicity of infection (MOI) of 1 for K562 and MEG.01 cells and at an MOI of 50 for HSPCs, obtaining a similar efficacy of transduction (Figure S5). Their EGFP expression kinetics (how the expression changes related to the expression of non-differentiated cells, referred as fold expression and detailed in Figures S3 and S4) were compared to that of endogenous WASP (filled bars in Figures 3C–3E) before and after MK differentiation. We observed similar behaviors of the three LVs, and all followed WASP expression kinetics upon MK differentiation in K562 cells (Figure 3C; upregulation), MEG-01 cells (Figure 3D; downregulation), and HSPCs (Figure 3E; downregulation). Interestingly, in the HSPC model, the kinetics of transgene expression at different times of MK differentiation were also very similar (Figure 3E).

**Figure 4:**
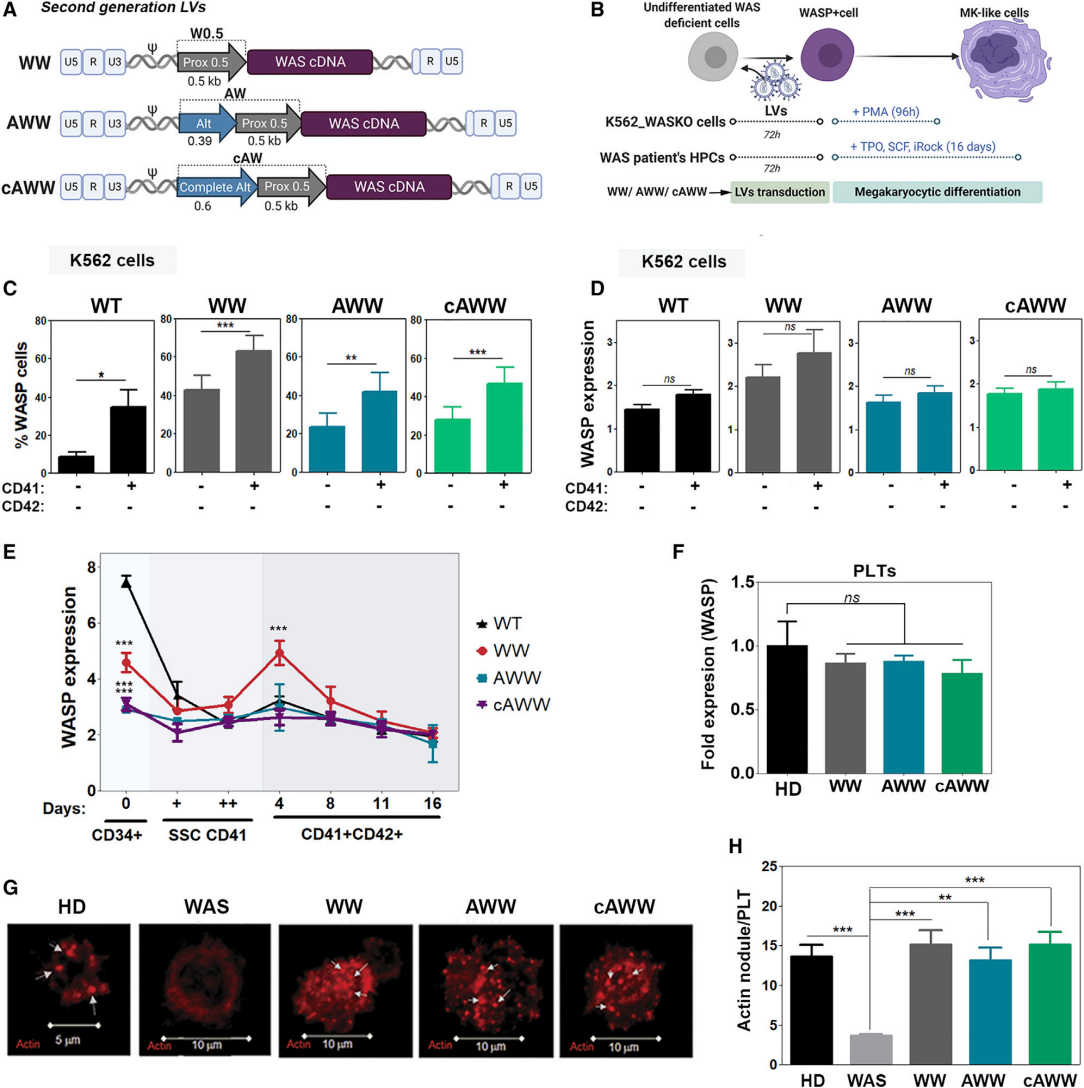
*WAS* Promoter-Driven LVs Harboring Sequences from the Alternative Promoter Rescue WASP Expression Kinetics in Human Cellular Models of WAS (A) Schematic representation of second-generation LVs expressing *WAS* cDNA. WW^21^ harbors the 500-bp core of the *WAS* proximal promoter, AWW,^26^ and cAWW (the present study). LVs contain an additional 386- and 600-bp fragment of the *WAS* alternative promoter, respectively (see Figure S2 for details). (B) Experimental diagram of K562 WASKO transduced with WAS LVs (MOI of 1) and differentiated to MK-like cells with PMA stimulation. (C and D) Graphs showing the percentage of WASP^+^ cells (C) and WASP expression levels (D) in WT K562 cells (black bars) and in WASKO K562 cells transduced with WW (gray bars), AWW (blue bars), and cAWW (green bars) after MK differentiation with PMA in CD41^−^CD42^−^ and CD41^+^ populations (n = 7). (E) Graph shows relative WASP expression in HD CD34^+^ cells and WAS patient cells transduced with WW (gray), AWW (blue), and cAWW (green) (MOI of 50) at 4, 8, 11, and 16 days of MK *in vitro* differentiation (n = 4). ∗∗∗p < 0.001, two-way ANOVA, Bonferroni post-test (compared to same day and conditions of HD). (F) Graph showing WASP expression levels of PLTs derived from WAS patient’s HSPCs transduced with WW, AWW, and cAWW related to WT-driven WASP expression. (G) Representative confocal images of actin nodule formation of platelets derived from HD CD34^+^ cells (left), WAS patient cells (second left), and WAS patient cells transduced with WW (middle), AWW (second right), and cAWW (right). Several nodules are indicated with white arrows. (H) Quantification of actin nodule per PTL counted in images from (G). Photographs analyzed: HD, 31; WAS, 6; WW, 17; AWW, 26; cAWW, 22. Non-parametric Mann-Whitney test, two-tailed.

We next analyzed whether the physiological expression was maintained in the different *WAS* promoter-driven LVs expressing the *WAS* cDNA, i.e., WW, AWW, and cAWW LVs (Figure 4A). In this study, we used homozygous WASKO-K562 cells previously generated by our laboratory^20^ (Figures 4B–4D) and WAS-null HSPCs from a WAS patient (Figures 4F–4I). WASKO-K562 cells were transduced with the different therapeutic LVs at an MOI of 1 to reach similar transduction efficacies (as shown in Figure S5). WASP expression levels were analyzed in different populations (see Figure S6 for details) after MK differentiation. Similarly to EGFP LVs, all WASP LVs increased WASP expression upon MK differentiation, measured as the percentage of WASP^+^ cells (Figure 4C) and as Median of Fluorescence Intensity (MeFI) (Figure 4D). However, the WW LVs showed higher WASP expression in resting K562 cells (Figure 4D, WW) compared to endogenous WASP (Figure 4D, wild-type [WT]), AWW, and cAWW LVs, indicating a more physiological behavior of the AWW and cAWW LVs.

Interestingly, AWW- and cAWW-transduced WAS patient HSPCs also recovered a WASP expression kinetic more similar to HD HSPCs compared to those transduced with the WW LVs (Figures 4E and S7). Indeed, although the expression levels of WW LVs in undifferentiated CD34^+^ were closer to those of HDs compared to AWW and cAWW (Figures 4E and 4F), the expression kinetic at early days of differentiation (day 4) were different (Figure 4E, red line). Nevertheless, WASP expression levels in PLTs derived from WAS patient HSPCs transduced with the different LVs (MOI of 50) were similarly rescued (Figure 4F). In agreement with WASP expression levels, the restoration of actin nodule^33^ formation in contact to immobilized fibrinogen, which is severely compromised in patient PLTs, was also similar for all LVs (Figures 4G and 4H).

### Phenotypic Correction of WASKO Mice after Transplantation with WW- and AWW-Transduced WASKO Murine HSPCs (mHSPCs)

**Figure 5:**
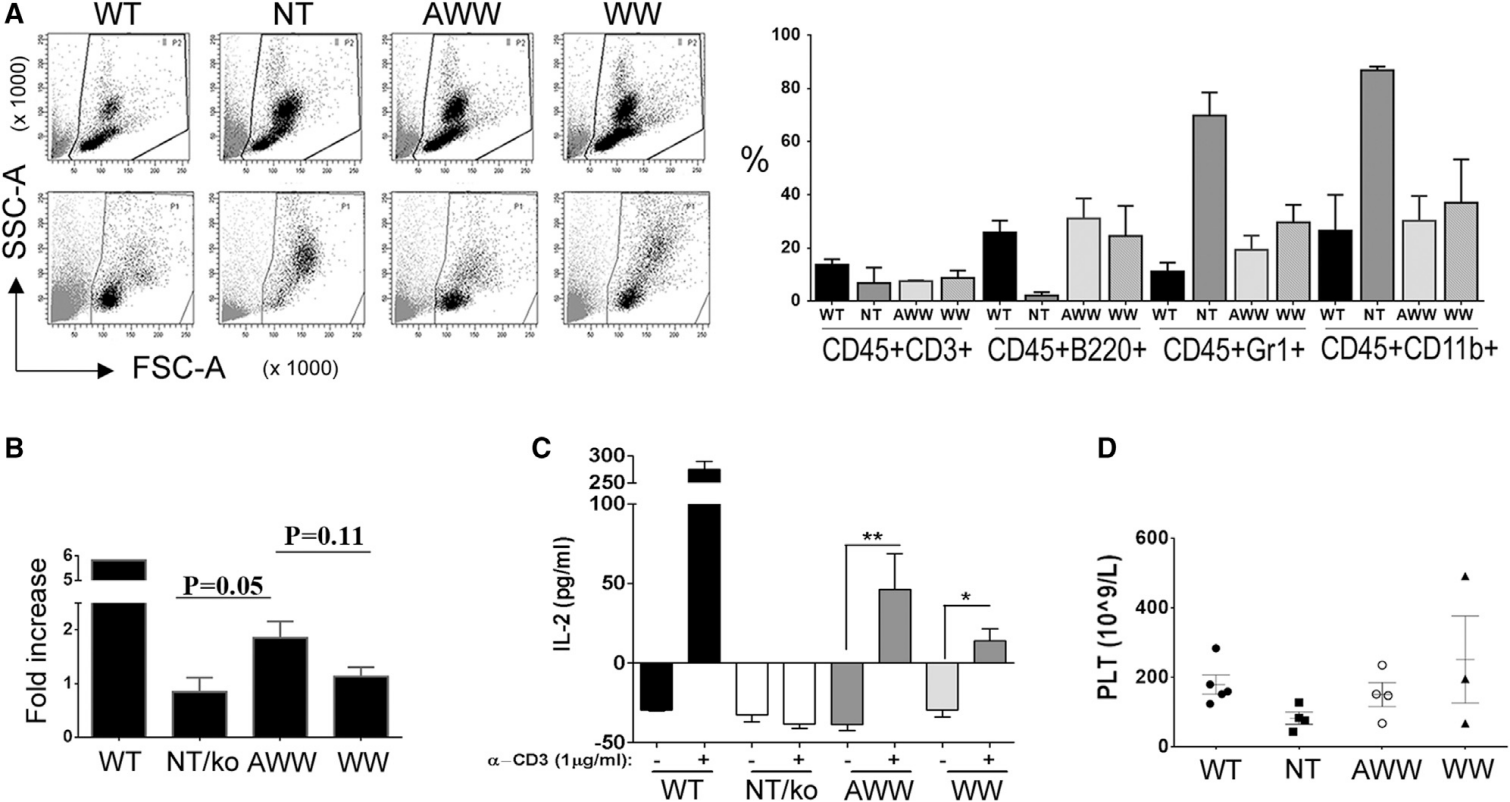
WW- and AWW-Transduced WASKO mHSPCs Rescue Phenotypic Defects of WASKO Mice (A) Restoration of the hematopoietic profile in mice transplanted with WW- and AWW-transduced WASKO mHSPCs (MOI of 100; WW mHSPCs = 8.2 vcn/c and AWW = 9.8 vcn/c). Side and forward scatter (left) of cells from PB of the transplanted mice 2 (left, up) and 6 (left, down) months after transplant. The non-transduced (NT) transplanted mice showed a higher number of granulocytes and a lower number of lymphocytes; meanwhile, the WT mHSPC and WASKO mHSPC AWW- and WW-corrected mice showed a normal distribution of the population in PB. 6 months post-transplant (right graph), mice were sacrificed and PB was analyzed for vcn/v (see Figure S9), CD45.2 (donor cells), and lineage markers CD3 (T cells), CD220 (B cells), Gr1^+^ (granulocytes), and CD11b (myeloid cells). Mice per group: WT = 2, NT = 2, AWW = 3, WW0.5 = 3. ∗p < 0.05, by unpaired t test. Data represent mean ± SEM. (B–D) Phenotypic rescue of WASKO mice after transplantation with WW- and AWW-transduced WASKO mHSPCs (MOI of 100; WW mHSPCs = 8 vcn/c and AWW = 6 vcn/c). (B and C) Mice were sacrificed after 3 months, and T cell proliferation assay (CFSE) (B) and IL-2 production (C) were measured after 5 or 3 days, respectively, after stimulation with anti-CD3 (1 μg/mL) by ELISA. One-tailed Mann-Whitney test. Data represent mean ± SEM (WT, N = 2; NT/KO, N = 2; AWW, N = 5; WW, N = 4). ∗p < 0.05, ∗∗p < 0.01. (D) Number of platelets in blood of transplanted mice compared with WT and NT (WT, N = 2, NT, N = 1, AWWN= 4, WW0.5 = 3). Copies/cell (cc) in spleen: AWW, 1.9 ± 1.1 cc; WW0.5, 1.8 ± 0.4 cc. Data represent mean ± SEM.

We next analyzed the therapeutic efficacy of AWW and WW LVs in a mouse model of WAS,^34^ because cAWW LVs did not show significant improvement *in vitro* compared with the AWW LVs. Lineage (Lin)^−^ cells were isolated from bone marrow of WASKO mice (WASKO mHSPCs) (see Materials and Methods) and transduced with AWW and WW LVs at an MOI of 100. Both LVs achieved similar transduction efficiencies (Lin^−^ AWW = 9.8 vector copy numbers/cell [vcn/c]; Lin^−^ WW = 8.2 vcn/c) and expressed similar WASP levels in WASKO mHSPCs (Figure S8B) and in their myeloid progeny (Figures S8E and S8F). Lethally irradiated WASKO mice (9.5 Gy) were then injected with 3 × 10^5^ AWW, WW, and mock WASKO mHSPCs as well as with WT mHSPCs (C57BL/6 mice). After 2 and 6 months of transplant, the SSC and FSC of cells from peripheral blood of the transplanted mice were analyzed (Figure 5A, left). The non-transduced transplanted mice showed a higher number of granulocytes and lower number of lymphocytes; meanwhile, the WT, AWW, and WW0.5 corrected mice showed a normal distribution of the population in peripheral blood. 6 months later, treated mice were sacrificed in order to analyze the ability of WW and AWW LVs to restore phenotypic and functional defects (Figure 5). Vector copy numbers per cell in spleen, bone marrow, and blood of transplanted mice ranged from 0.1 to 15, showing efficient repopulation of recipient mice with transduced AWW and WW Lin^−^ cells (Figure S9A). Both LVs were equally efficient in restoring normal monocyte counts in a hemogram (Figure S9). More importantly, transplanted mice achieved equivalent ratios of B220^+^, Gr1^+^, and CD11b^+^ cells (Figure 5A, right) as compared to WT mice, whereas WASKO mice presented reduced levels of B220^+^ cells and increased levels of Gr1^+^ and CD11b^+^ cells. Similarly, the efficacy of both LVs to rescue T cell responses (Figures 5B and 5C) and the PLT counts (Figure 5D) were equivalent. These experiments validate the efficacy of both *WAS* promoter-driven LVs as tools for WAS GT, but they cannot differentiate any superiority.

### Third-Generation *WAS*-Driven LVs Have Improved Physiological Expression and Restore Functional Defects *In Vitro* and *In Vivo*

**Figure 6:**
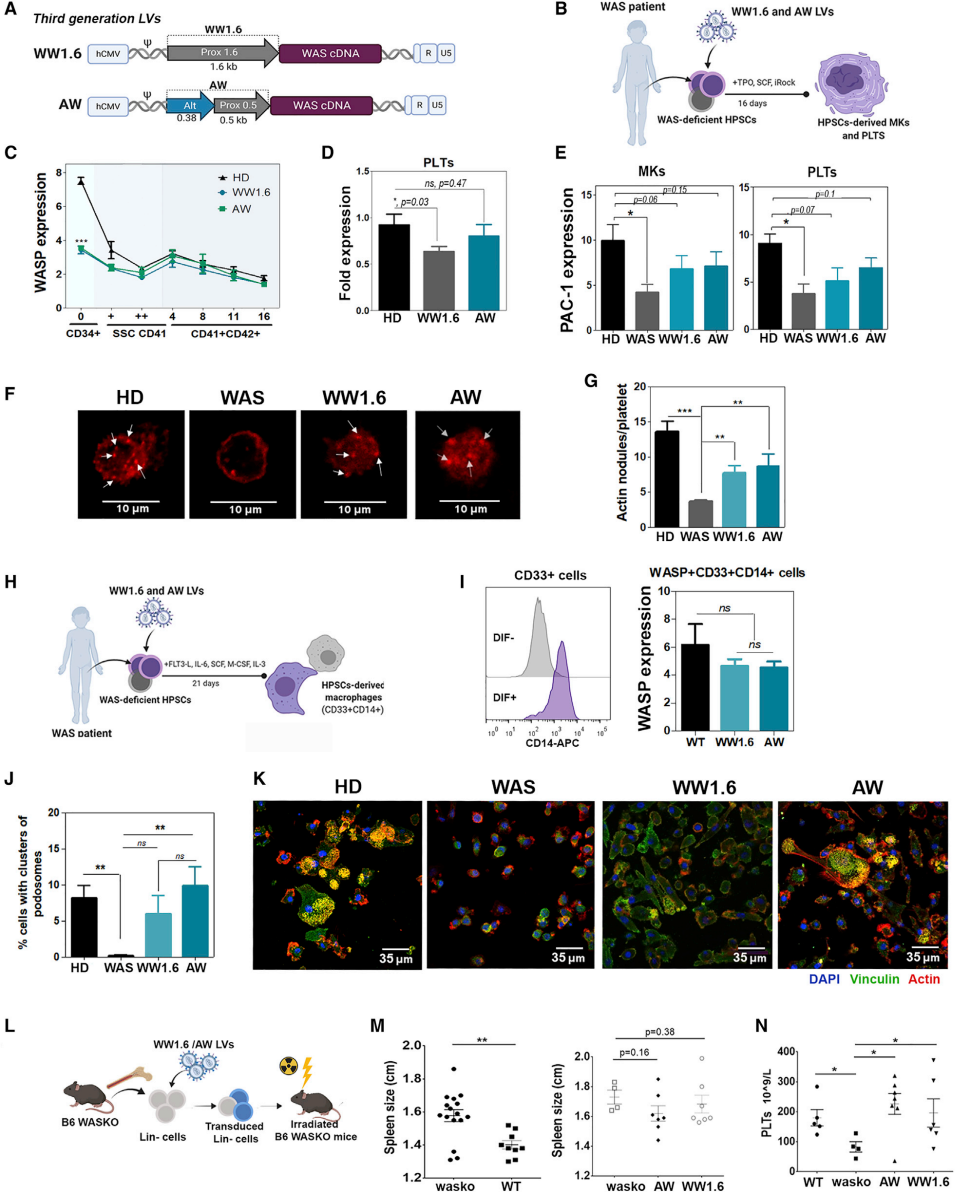
Third-Generation *WAS*-Driven LVs Mimic Endogenous WASP Expression Kinetics during MK Differentiation and Restore Functional Defects *In Vitro* and *In Vivo* (A) Third-generation LVs used for the study. WW1.6 referred the current clinical vector hWW1.6 that includes the complete *WAS* 1.6-kb proximal promoter^15^ (AC115618.3 Seq 120189–121855) and AW that includes an alternative promoter previously described (AC115618.3: Seq 114605–114991/17 bp/ Seq 121356–121851).^26^ (B) HSPCs from a WAS patient were transduced with the third-generation LVs at an MOI of 50 (WW1.6 = 2.61 ± 1.13 vcn/c and AW = 3.16 ± 1.61 vcn/c), and WASP expression and functional restoring were analyzed after in *vitro* MK differentiation. (C) Relative WASP expression of HD, WAS_WW1.6, and WAS_AW in the CD41^+^CD42^+^ gated population at days 4, 8, 11, and 16 of MK differentiation. Represented data are means ± SEM. Mann-Whitney test, two-tailed, ∗∗∗p < 0.001 (n = 5). (D) WASP relative expression levels in PLTs (CD41^+^CD42^+^FSC^low^SSC^low^) derived from HD, WAS_WW1.6, and WAS_AW HSPCs compared to those of HD-derived PLTs. ∗p < 0.05 (n = 4). (E) PAC-1 expression of gated MKs (CD42^+^FSC^high^SSC^high^) (left) and PLTs (CD42^+^FSC^low^SSC^low^) (right) from HD-, WAS-, WAS_WW1.6-, and WAS_AW-derived HSPCs. Represented data are means ± SEM. Mann-Whitney test, two-tailed, ∗p < 0.05 (n = 3). (F) Confocal images of actin nodules in platelets derived from HSPCs. White arrows indicate examples of actin nodules. (G) Quantification of actin nodules/platelet in the transduced cell photographs analyzed; ∗∗p < 0.01, ∗∗∗p < 0.001. HD = 31, WAS = 6; WW1.6 = 5, AW = 9. Mann-Whitney test, two-tailed. (H) Schematic diagram showing the procedure to study podosome restoration in monocytes derived from WAS patient’s HPSCs. (I) Histograms showing CD14 expression after monocytic differentiation (DIF^+^) in the CD33^+^ population (left). WASP expression (right) was determined in the CD33^+^CD14^+^ population derived from healthy (black bars), WAS patient cells (set as control, not shown), and WAS patient cells transduced with WW1.6 (light-blue bars) and AW (dark-blue bars) LVs at the end of the differentiation (day 20). Non-parametric Mann-Whitney t test, not significant (WT, N = 3; WW1.6 and AW, N = 4). (J) Graph shows the percentage of cells with podosomes in a cluster structure (composed for at least 20 podosomes). Photographs from three different experiments were analyzed: HD (18 photographs, 338 cells), WAS patient (12 photographs, 195 cells), WW1.6 (12 photographs, 110 cells), AW (14 photographs, 198 cells). Non-parametric Mann-Whitney t test, two-tailed, ∗∗p < 0.05. (K) Representative images of podosomes in clusters from confocal microscopy. Cells obtained *in vitro* were incubated overnight onto fibronectin-coated chambers at 37°C, and nuclei (DAPI, blue), actin (red), and vinculin (green) were stained. (L) Schematic diagram of transplant procedure of WASKO mice with AW- and WW1.6 Lin^−^ WASKO-transduced cells (Lin^−^ AW = 5 vcn/c, Lin^−^ WW1.6 = 8 vcn/c). (M) Graphs showing spleen size (longitudinal size measured in centimeters) of WT and WASKO mice (mice 10–12 weeks old, left graph) and WASKO mice transplanted with AW- and WW1.6-transduced Lin^−^ WASKO cells (18–20 weeks old, right graph). (N) Graph showing PLT counts in WT and WASKO mice as well as in WASKO mice transplanted with Lin^−^ WASKO cells transduced with AW and WW1.6 LVs. Vector copies analyzed in spleen: AW = 1.03 ± 0.31 vcn/c and WW1.6 = 0.58 ± 0.30 vcn/c.

We finally generated third-generation LVs based on the AWW and investigated their behavior during MK differentiation as well as their therapeutic activity in WASKO mice. We used the WW1.6 LV^15^ (Figure 6A, top; this is the LV used in ongoing clinical trials for WAS GT) as the backbone to construct the third-generation AW LV by replacing the full-length (1.6 kb) *WAS* proximal promoter by the chimeric *WAS* promoter (0.88 kb) containing sequences from the proximal and alternative promoter (Figures 6A and S2). WAS patient HSPCs were transduced with WW1.6 and AW LVs at an MOI of 50 to achieve similar transduction efficacies (WW1.6 = 2.6 ± 1.1 vcn and AW = 3.1 ± 1.6 vcn and Figure S5F). Transduced cells were differentiated into MKs and the different subpopulations were analyzed for WAS expression (Figures 6B and S9). As in second-generation LVs, the WASP expression dropped during MK differentiation in both LVs (Figure 6C). We could not find any differences between both LVs in either terms of expression kinetics (Figures 6C) or in PLT expression levels (Figure 6D). However, a comparison of the WASP expression levels of each vector with WASP expression in PLTs from HDs showed significant differences with PLTs derived from 1.6WW-transduced HPSCs, but not with PLTs derived from AW-transduced HPSCs, although more experiments are necessary to demonstrate this. We next evaluated the functional restoration of MKs and PLTS derived from WW1.6- and AW-transduced WAS HSPCs by measuring PAC-1 expression in response to thrombin (Figures 6E), the formation of actin nodules (Figures 6F and 6G), or calcium uptake (Figure S11). We found a very similar improvement with both LVs, restoring the response of MKs and PLTs to thrombin, as well as the ability of PLTs to form actin nodules per PLT. Similar findings were also observed for the restoration of podosome formation and clustering on macrophages derived from WAS HSPCs transduced with WW1.6 and AW LVs (Figures 6H–6K), which exhibited similar WASP expression in the CD33^+^CD14^+^ cells obtained *in vitro* (Figure 6I). We finally analyzed the therapeutic efficacy of WW1.6 and AW LVs in the WASKO mouse model. WASKO mHSPCs were isolated from bone marrow, transduced with both LVs, and transplanted into irradiated WASKO mice (Figure 6l). Mock-transduced WASKO mHSPCs and WT mHSPCs were injected into control mice. 7 months later, treated mice were sacrificed to analyze the ability of WW1.6 and 0.88-kb AW (AW0.88kb) to restore functional defects. Both LVs were equally efficient in restoring spleen size (Figure 6M) and PLT counts (Figure 6N).

## Discussion

GT has demonstrated to be a real therapeutic alternative for WAS patients.^7^^,^^11^, ^12^, ^13^, ^14^ The current GT approaches for WAS used autologous HSPCs (CD34^+^) genetically modified using a 1.6-kb *WAS*-proximal promoter-driven LV and transplanted back into the patients. The clinical efficacy of these LVs has been clearly demonstrated since treated patients have shown a reduction of infections and diminished severity of eczema as well as reduced frequency and severity of bleeding episodes. However, although the bleeding was reduced or completely corrected in most patients,^7^^,^^14^ microthrombocytopenia persisted and normalized PLT counts were not achieved in most patients.^11^^,^^14^^,^^16^ The reasons behind the low PLT recovery achieved with GT compared with the other immune functions are unclear. It was postulated that PLT recovery could correlate with the number of transduced HSPCs reinfused. In this case, the absence of a potent selective advantage in MKs and MK progenitors expressing WASP could be a contributory factor in contrast to lymphocytic lineages.^17^ Other hypotheses are that the WW1.6 LV achieved suboptimal levels of WASP expression per copy of integrated vector in MKs, MK progenitors, and/or PLTs.^14^ At the same level of HPC transduction, it may be possible to correct the function of lymphoid cells but not that of MK lineage cells. Furthermore, PLTs with suboptimal WASP levels could undergo accelerated destruction in the spleen and in other organs.

An ideal GT vector for WAS should not only transduce HSPCs efficiently, but also mimic endogenous WASP expression during HSPC differentiation to the different hematopoietic lineages. Our hypothesis is that physiological expression of WASP during MK differentiation and in PLTs would improve GT outcomes. The aim of this work was therefore to investigate WASP expression along MK differentiation and to define which therapeutic LV mimics more closely represent this pattern.

Although WASP expression levels are well established in most mature blood cells,^30^^,^^31^ the WASP expression kinetics during MK differentiation and thrombopoiesis are not largely studied. We used immortalized cellular models (K562 and MEG-01) as well as primary HSPCs to investigate WASP expression kinetics during these processes. Our data showed an initial decrease in WASP expression from HSPCs to MK progenitors, a slight increase when they are differentiated into early MKs, and a gradual downregulation as these MKs mature into PLTs. This WASP expression kinetic has never been described before and it could be of relevance for normal MK differentiation and thrombopoiesis. We corroborated this expression pattern on two immortalized cell lines, K562 and MEG-01, that resemble different stages of MK differentiation. K562 cells have been described previously as a multipotent myeloid-MK cell line for the study of early events in MK differentiation,^35^^,^^36^ while MEG-01 cells are defined as an immature MK cell line able to differentiate into mature MKs and PLTs.^37^^,^^38^ Interestingly, upon MK differentiation, K562 cells mimicked the differentiation phase from MK progenitors into early MKs, increasing WASP levels, while MEG-01 cells mimic the differentiation stage from early MKs into mature MKs and PLTs, reducing WASP expression. The exact role of these changes in WASP expression during MK differentiation is not clear; however, because the absence of WASP increases PLT production with abnormal phenotype,^18^, ^19^, ^20^ it could be relevant for GT strategies to mimic it. Therefore, our hypothesis is that WAS GT strategies should not only achieve good WASP expression levels on PLTs and MK differentiated cells but also mimic the endogenous expression kinetic during MK differentiation.

Once we had established the physiological WASP expression pattern during megakaryopoiesis and thrombopoiesis, we analyzed the behavior of different LVs harboring different fragments of the *WAS* proximal and alternative promoters. Our previous data indicated that an LV driving EGFP through a *WAS* promoter (AWE), containing a 386-bp fragment of the alternative promoter immediately upstream of the 500-bp *WAS*-proximal promoter, increased GFP expression in myeloid cells and mature MKs.^26^ The alternative promoter is located 6 kb upstream of the proximal promoter and contains several transcription factor binding sites (TFBSs) shared with the proximal promoter (Sp-1, AP-2, c-Myb, and EGR2) and others that are unique (C/EBP, CP1, Ets-2, and GCF).^28^^,^^39^ The differences found between both promoters suggest that their activity may vary depending on the cell lineage and the state of differentiation or development. In particular, the presence of TFBSs for C/EBP, CP1, c-Myb, Ets-2, and PU.1 suggest that the alternative promoter could play important roles in the myeloid and megakaryocytic lineages.^40^, ^41^, ^42^ However, the 386-bp fragment of the alternative promoter does not include binding sites for C/EBP and CP1, which are required for higher expression levels in K562 cells.^39^ Based on these data, we generated new *WAS*-driven LVs (cAWE and cAWW) harboring the complete alternative promoter and the 500-bp proximal promoter and compared their behavior with the *WAS*-proximal promoter LVs (WE and WW), the shortest version of the alternative promoter (386 bp), and the 500-bp proximal promoter (AWE and AWW). The different second-generation LV backbones (W-, AW-, and cAW-) were studied based on their ability to express *EGFP* and *WAS* cDNA during MK differentiation and analyzing whether this expression followed the same kinetic as that of the endogenous WAS protein. Although all EGFP-expressing LVs mimicked the WASP expression pattern along MK differentiation, the WE LV was slightly weaker than the AWE and cAWE LVs. Interestingly, the expression of *WAS* cDNA altered the physiological behavior of the 500-bp *WAS*-proximal promoter LVs (WW) at early stages of MK differentiation, while the AWW and the cAWW LVs still followed the WASP endogenous pattern. Our finding indicates that the 500-bp *WAS*-proximal promoter-driven LVs lead to a WASP overexpression at early stages of MK differentiation (CD34^+^CD41^+^CD41^−^ to CD34^+^CD41^+^CD42^+^), while AW and cAWW achieve more physiological expression. A detailed analysis of WASP kinetics along MK differentiation revealed a more pronounced decay of WASP expression of the *WAS*-proximal promoter-driven LVs compared to the *WAS*-proximal and alternative promoters and to endogenous WASP. However, we could not observe significant differences in the behavior of AWW versus cAWW LVs. We therefore concluded that the insertion of regulatory sequences from the *WAS* alternative promoter into the 500-bp proximal promoter improved physiological expression of therapeutic LVs during MK development.

Despite their improved physiological expression, we could not detect significant differences between the second-generation WW and AWW LVs in terms of functional correction either in animal models or in the HSPCs of WAS patients. Importantly, our analysis showed that LVs expressing the WAS cDNA through the proximal and alternative promoter (in a second- or third-generation backbone) are able to (1) restore actin nodules in PLTs (Figures 4G and 4H); (2) normalize WAS mice blood populations upon transplantation with transduced WASKO Lin^−^ cells (Figures 5A and S9); (3) improve T cell responses (Figures 5B and 5C); (4) improve PLT counts (Figures 5D and 6N); (5) reduce spleen size (Figure 6M); (6) restore podosome clustering of macrophages derived from transduced WAS-null human hHSPCs (hHSPCs) (Figures 6H–6K); and (7) restore calcium uptake dynamics after thrombin stimulation of PLTs derived from transduced WAS-null hHSPCs (Figure S11).

Still, since the AWW LVs confer a more physiological expression profile during MK differentiation, we reasoned that it would be relevant to investigate the potential of third-generation LVs harboring the shorter version of the alternative and proximal promoter (AW) as another option to the existing therapeutic LV for WAS GT, i.e., WW1.6, which drives the expression of *WAS* cDNA through a 1.6-kb fragment of the *WAS*-proximal promoter. Contrary to what was found with the LVs harboring the 0.5-kb proximal promoter, the LV used in the ongoing clinical trials for WAS, i.e., the WW1.6 LV, was as good as the AW mimicking the WASP endogenous pattern. We finally showed a very similar therapeutic efficacy of both third-generation LVs WW1.6 and AW in WAS patient HSPCs as well as in WAS mouse models. These data indicate that the thrombocytopenia defects found in WW1.6 LV-treated patients is not due non-physiological WASP expression kinetics, and they favor the hypotheses of low numbers of engrafted WASP+HSPCs. In addition, we cannot completely exclude that WASP expression levels are inadequate for *in vivo* production of PLTs in humans, since our *in vitro* models may not fully model these steps.

In summary, in this work we have identified the WASP expression kinetics during MK differentiation, which show an initial decrease in WASP expression as HSPCs differentiate into MK progenitors, a slight increase when progenitors differentiate into early MKs, and a continuous downregulation as these MKs mature into PLTs. Based on these data, we have shown that the 500-bp *WAS*-proximal promoter-driven LVs improved their physiological behavior after inclusion of regulatory sequences from the alternative promoter. However, the WW1.6 LV was as good as the AW in mimicking the WASP expression pattern and also in restoring functional defects of HSPCs in WAS patients and WASKO mice models. Collectively, our data indicated that the WW1.6 and AW LVs are able to mimic WASP endogenous expression patterns during MK differentiation. We propose to use the AW LVs for a clinical trial for WAS GT in order to investigate potential therapeutic benefits over the WW1.6 LVs.

## Materials and Methods

### Cells

293T cells (CRL11268; American Type Culture Collection, Rockville, MD, USA) were maintained in Dulbecco’s modified Eagle’s medium (DMEM, Thermo Fisher Scientific, Waltham, MA, USA) with GlutaMAX supplemented with 10% heat-inactivated fetal bovine serum (FBS, Sigma-Aldrich, St. Louis, MO, USA) and antibiotics. The human cell line K562 (lymphoblasts from bone marrow chronic myelogenous leukemia [CML]) was obtained from ATCC (CCL-243) and maintained in RPMI 1640 media (Invitrogen) supplemented with 10% FBS at 5% CO_2_ and 37°C. Autologous CD34^+^ cells were collected from mobilized peripheral blood (after cryopreservation of an unmodified backup stem cell harvest). The WAS patient carried the *WAS* mutation c.58C>Tp.(Gln20∗). Cells were grown for 6 days in StemSpan media (STEMCELL Technologies, Vancouver, BC, Canada) supplemented with 1% penicillin/streptomycin, 100 ng/mL recombinant human Stem Cell Factor (SCF), 100 ng/mL *fms*-related tyrosine kinase 3 ligand (Flt-3L), 20 ng/mL thyroperoxidase (TPO), 20 ng/mL interleukin (IL)-6 (all from PeproTech, NJ, USA), 1 μM StemRegenin 1 (SR1) (Cayman Chemical, MI, USA), 500 nM UM729 or UM171 (STEMCELL Technologies), and 10 μM 16,16-dimethyl prostaglandin E_2_ (dmPGE2) (Cayman Chemical). All current regulations have been complied for the experimentation with patient samples, following the current Spanish legal regulations on research with humans (Real Decreto 561/1993 of April 16 (BOE 1993; no For usage of human CD34+ HSPCs from HDs and WAS patient, informed written consent was obtained in accordance with the Declaration of Helsinki and ethical approval from the Great Ormond Street Hospital for Children NHS Foundation Trust and the Institute of Child Health Research Ethics (08/H0713/87).

Bone marrows of C57BL/6J and B6.129S6-*Was*^*tm1Sbs*^/J mice were harvested from the femurs and tibias, and Lin^−^ progenitors were isolated with magnetic beads using a lineage cell depletion kit (130-090-858, MACS, Miltenyi Biotec, Germany) following the manufacturer’s instructions. 1 × 10^6^ cells/mL were cultured in StemSpan media (STEMCELL Technologies) supplemented with 1% penicillin/streptomycin, 1% glutamine, 100 ng/mL murine (m)SCF, 20 ng/mL mFlt-3L, 20 ng/mL mIL-3 and 20 ng/mL mIL-6 (PeproTech).

Human PLTs were obtained by centrifugation of peripheral blood at 200 × *g* for 20 min. The supernatants were recovered and centrifuged at 1,000 × *g* for 10 min in the presence of prostacyclin I2 (0.1 μg/mL, Abcam ab120912). The pellet was resuspended in modified Tyrode’s buffer (TB; 150 mM NaCl, 2.9 mM KCl, 12 mM NaHCO_3_, 0.1% glucose, 0.1% BSA, 5 mM HEPES, 1 mM CaCl_2_, and 1 mM MgCl_2_ [pH 7.3]) containing 0.1 μg/mL prostacyclin.

### Animals

C57BL/6J and B6.129S6-*Was*^*tm1Sbs*^/J mouse colonies were already established at the animal facility of University College London (UCL) (original stock obtained from The Jackson Laboratory, USA). NOD.Cg-*Prkdc*^*scid*^ *Il2rg*^*tm1Wjl*^/SzJ mice were obtained from The Jackson Laboratory (USA). All animals were handled in strict accordance with good animal practice as defined by UK Home Office Animal Welfare Legislation, and all animal work was approved by the Institutional Research Ethics Committee (Institute of Child Health, University College London, UK) and performed under project license nos. 70/7024 and 2557.

### LV Plasmid Constructs

WW and WE carry a 0.5-kb fragment of the *WAS*-proximal promoter (GenBank: AC115618.3 Seq 121356–121851) driving the expression of human *WAS*^21^ and *EGFP* cDNAs, respectively. AWW and AWE were engineered by inserting a 0.38-kb fragment of the *WAS* alternative promoter^22^ immediately upstream of the 0.5-kb *WAS* proximal promoter in the WW vector^26^ (GenBank: AC115618.3: Seq 114605–114991 linker of 17-nt fragment containing the EcoRI site and Seq 121356–121851) driving the expression of human *WAS* and *EGFP*, respectively. cAWE contains the complete alternative promoter (0.69 kb) upstream of the 500-bp proximal promoter (GenBank: AC115618.3: Seq 114403–115000 linker of 17-nt fragment containing the EcoRI site Seq 121356–121851) for the expression of EGFP. The cAWW plasmid was obtained after insertion of *WAS* cDNA in the cAWE backbone (instead of the EGFP sequence) (restriction enzymes BamHI and KpnI) (New England Biolabs), by standard cloning techniques. All of these LVs share the SIN lentiviral backbone described by Zuffery et al.^43^

For the construction of the third-generation AW LVs, we used the WW1.6 plasmid^15^ (kindly provided by Généthon) as backbone and replaced the ClaI/BstXI fragment containing the 1.6-kb *WAS*-proximal promoter (GenBank: AC115618.3 Seq 120189–121855), by the ClaI/BstXI fragment from the AWW LV^26^ harboring the *WAS* alternative and proximal promoter (0.38+0.5 kb) (GenBank: AC115618.3: Seq 114605–114991 a linker of a linker of 17-nt fragment containing the EcoRI site Seq 121356–121851).

### LV Production, Titration, and MOI

LV particles were produced by polyethylenimine (PEI) (Sigma-Aldrich, no. 408727) or lipoD293 (SignaGen Laboratories, Gaithersburg, MD, USA) as previously described.^44^ Briefly, for second-generation LVs, 293T packaging cells were transfected with packaging (pCMVΔR8.91), envelope (pMD2.G) (http://www.addgene.org/Didier_Trono/) , and the desired vector plasmids (WW, WE, AWW, AWE, cAWW or cAWE). The third-generation LVs were obtained by the following four transfection plasmids: CTE (gag/pol), pMD2.G (envelope), pRev plasmids, and vector plasmid (AW and WW1.6). The producer cells were cultured for 48 and 72 h, and the viral supernatants were collected at those times and filtered through a 0.45-μm filter (Stericup and Steritop sterile filters, Merck Millipore, MA, USA). The viral particles were then concentrated by ultracentrifugation in a Sorvall Discovery SE ultracentrifuge (Kendro Laboratory, Weaverville, NC, USA) at 40,000 rpm for 2 h at 4°C, and the viral pellets were resuspended in StemSpan media (STEMCELL Technologies) for 1 h on ice, aliquoted, and immediately frozen at −80°C.

Viral titers (transduction units [TU]/mL) were calculated using quantitative PCR. Briefly, 293T cells were transduced with serially diluted amounts of LV. Genomic DNA was isolated (10^5^ cells equivalent to 0.6 μg of genomic DNA) and the copy number of LVs integrated was measured using a standard curve (from 10^2^ to 10^7^ copies) of plasmid DNA. Titers obtained for the therapeutic LVs were as follows: WW, 7.91 × 10^7^ to 6.35 × 10^9^ TU/mL; AWW, 1.58 × 10^8^ to 8.67 × 10^9^ TU/mL; cAWW, 1.07 × 10^8^ to 7.24 × 10^9^ TU/mL; WW1.6, 1.13 × 10^9^ to 3.47 × 10^9^ TU/mL; AW, 6.52 × 10^8^ to 3.31 × 10^9^ TU/mL.

Immortalized cell lines and primary hHSPCs were incubated with the different viral supernatants for 5 h at 37°C 5% CO_2_. Transduction levels of the cells were determined by flow cytometry 48–72 h after transduction.

A different MOI was established for the different cell types. K562 and MEG-01 cells were transduced at an MOI of 1, hHSPCs at an MOI of 50, and mouse HSPCs at an MOI of 100.

### Megakaryocytic Differentiation of K562 and MEG-01 Cell Lines

10^5^ K562 cells/mL were plated on a six-well plate and incubated with 30 nM of PMA (phorbol 12-myristate 13-acetate, Sigma-Aldrich) during 96 h without media exchange. DMSO was added as negative control of differentiation. 10^5^ MEG-01 cells/mL were incubated with 2 mM VPA (Sigma-Aldrich) during 21 days. Media were changed every 2–3 days with fresh VPA. Cells were detached with TrypLE (5 min, 37°C) for flow cytometry analysis.

### Megakaryocytic Differentiation of HSPCs (CD34^+^)

2.5 × 10^5^ cells/well were seeded in a 24-well plate in a final volume of 1 mL of StemSpan serum-free expansion medium (SFEM) (STEMCELL Technologies) supplemented with TPO (50 ng/mL) and SCF (5ng/ml) and added to the cells on days 0, 4, 8, 11, and 16. The concentration of the cells was always kept below 1 × 10^6^ cells/mL. Inhibitor of the kinase ROCK (iROCK) was added to the medium previously described at day 8 of differentiation to a final concentration of 10 μM. Cells were harvested at different time points for staining with anti-human CD34-phycoerythrin (PE)-Cy7, anti-human CD41a-PE, and anti-human CD42b-allophycocyanin (APC) (all from eBioscience) and analyzed by flow cytometry.

### Activation Assay: Calcium Flux

MKs and PLTs derived from HSPCs were washed at 900 × *g* during 5 min and resuspended in TB (Sigma). Cells were stained with CD42b-APC during 20 min at room temperature (RT) and washed with TB. Then, cells were incubated with calcium-eFluor 514 (eBioscience) at a final concentration of 5 μM during 40 min at 37°C. Cells were washed in TB and acquired on a FACsCanto II flow cytometer during 30 s to establish basal levels (t = 0). Then, thrombin (2 U/mL) was added and acquired immediately during 90 s.

### Activation Assay: Binding of PAC-1

MKs and PLTs from HSPCs were centrifuged at 900 × *g*, washed, and resuspended in TB with calcium and magnesium. Cells were activated with thrombin (2 U/mL) during 10 min at RT and stained anti CD42b-APC and anti PAC-1-fluorescein isothiocyanate (FITC) (all from eBioscience) and incubated during 25 min at RT. The cell suspension was washed with TB, fixed with 0.25% paraformaldehyde (PFA) in TB, and acquired on a FACSCanto II flow cytometer.

### Actin Nodule Formation Assay

MKs and PLTs derived from HSPCs were harvested and washed in TB in the presence of prostacyclin I2 (Abcam, Cambridge, UK). Cells were seeded in chamber slides (Lab-Tek II chamber slide, Thermo Fisher Scientific) pre-treated with fibrinogen (10 μ/mL, Sigma-Aldrich), and they were left to adhere for 30 min at 37°C without stimuli. Chambers were washed briefly with PBS and fixed with 10% formalin for 10 min at RT. After three washes with PBS, fixed cells were treated with 50 mM NH_4_Cl for 10 min for quenching residual fluorescence, washed three times, and permeabilized with 0.1% Triton X-100 for 10 min. After washing, cells were incubated with rhodamine-phalloidin (1:200, Thermo Fisher Scientific) during 30 min, washed, and mounted with ProLong Gold antifade reagent (Thermo Fisher Scientific). Images were acquired on a Confocal Laser Zeiss LSM 710 microscopy and analyzed with ImageJ (National Institutes of Health [NIH], Bethesda, MD, USA). Actin nodules per PLT were counted for WT (31 images), WAS patient (6), WW (17), AWW (26), cAWW (22), WW1.6 (4), and AW (9) in three independent experiments.

### Myeloid Differentiation of HSPCs (CD34^+^)

Briefly, 50,000 HSPCs were cultured in low-attachment 24-well plates with StemSpan (STEMCELL Technologies) supplemented with Flt-3L (50 ng/mL), SCF (200 ng/mL), IL-3 (10 ng/mL), IL-6 (50 ng/mL), and macrophage colony stimulating factor (M-CSF) (50 ng/mL) (all from PeproTech) during 21 days. Media were renewed every 2–3 days. Monocytic differentiation was determined by the increment of FSC and SSC and expression of CD33-PE and CD14-APC (all from eBioscience) by flow cytometry.

### Podosome Immunostaining

200,000 HSPC derived-macrophages were cultured overnight over Lab-Tek II chamber slides (Thermo Fisher Scientific) coated with 10 μg/mL fibronectin (Thermo Fisher Scientific). On the next day, cells were fixed with 4% formaldehyde, permeabilized with 0.1% Triton X-100 in PBS, and blocked with PBS+1% BSA. Then, cells were incubated with phalloidin-Alexa Fluor 568 (Thermo Fisher Scientific) (20 min) to detect F-actin, washed, and incubated with anti-vinculin antibody (hVIN-1) (Sigma-Aldrich, St. Louis, MO, USA) (20 min). After washing, the cells were incubated with a goat anti-mouse immunoglobulin G (IgG) conjugated to Alexa Fluor 488 (Invitrogen) and DAPI, and they were placed on slides with mounting media (ProLong Gold, Thermo Fisher Scientific). Images were captured on a Zeiss LSM 710 inverted confocal microscope (Zeiss, Oberkochen, Germany). Images were processed in Fiji-ImageJ program (NIH, Bethesda, MD, USA). Clusters of podosomes were defined as a zone of at least 20 podosomes.

### Purification, Transduction, and Transplantation of Lin^−^ Cells

Bone marrow of C57BL/6J and B6.129S6-*Was*^*tm1Sbs*^/J mice was harvested from the femurs and tibias, and Lin^−^ progenitors were isolated with magnetic beads using a lineage cell depletion kit (130-090-858, MACS, Miltenyi Biotec, Germany) following the manufacturer’s instructions. 1 × 10^6^ cells/mL were cultured in StemSpan media (STEMCELL Technologies) supplemented with 1% fetal calf serum (FCS) (Invitrogen), 1% penicillin/streptomycin, 100 ng/mL murine SCF, 20 ng/mL mFlt-3L, 20 ng/mL mIL-3, and 20 ng/mL mIL-6. Isolated murine Lin^−^ cells were transduced for 16–18 h with the different LVs (MOI of 100). A sample was retained and further cultured for 72 h to determine transduction efficiency (integrations per cell), *in vitro* differentiation into myeloid or dendritic cells, and for transplantation into B6.129S6-*Was*^*tm1Sbs*^/J mice. WT, non-transduced, and transduced WASKO Lin^−^ cells (3 × 10^5^ to 1 × 10^6^) were inoculated intravenously into lethally irradiated mice (split dose of 9.5 Gy). Animals were sacrificed by using CO_2_ inhalation and cervical dislocation 3–7 months after transplants. Percentage of myeloid populations and expression of WASP was analyzed.

### LV Integrations in Transplanted WASKO Mice

Genomic DNA from spleens of transplanted WASKO mice was extracted using the DNeasy blood and tissue kit (QIAGEN, Hilden, Germany). The quantitative PCR was performed in a CFX96 Touch real-time PCR detection system (Bio-Rad, CA, USA). To amplify the vector, we used sequences annealing the human WAS described by Charrier et al.^45^ and HIV-psi sequences (forward, 5′-CAGGACTCGGCTTGCTGAAG-3′, reverse, 5′-TCCCCCGCTTAATACTGACG-3′, and probe, 5′-FAM-CGCACGGCAAGAGGCGAGG-TAMRA-3′). Titin was used as endogenous two-copy gene control (forward, 5′-AAAACGAGCAGTGACGTGAGC-3′, reverse, 5′-TTCAGTCATGCTGCTAGCGC-3′, and probe: 5′-FAM-TGCACGGAAGCGTCTCGTCTCAGTC-TAMRA-3′). Serially diluted plasmid DNA containing the relevant sequences was used as a standard curve, with all measurements performed in duplicate.

### Proliferation and IL-2 Production of Murine T Cells

Murine T cells were purified from spleen with a CD90.2 MicroBeads isolation kit (Miltenyi Biotec). For TCR stimulation, 96-well plates were coated with 1 μg/mL anti-CD3e (functional grade purified) (Affymetrix, eBioscience). CD90.2^+^ cells were preincubated with CFSE (5-(and 6)-carboxyfluorescein diacetate, succinimidyl ester) dye (CellTrace CFSE cell proliferation kit, Thermo Fisher Scientific) and plated in triplicate for 5 days in RPMI 1640 supplemented with 10% FCS and 1% penicillin/streptomycin. At day 3, supernatant from each well was saved for IL-2 analysis (DuoSet ELISA mouse IL-2, R&D System, Minneapolis, MN, USA) following the manufacturer’s instructions. At day 5, proliferation was measured by flow cytometry in a CyAn ADP analyzer (Beckman Coulter), and the analysis was performed with FlowJo software (Tree Star, Ashland, OR, USA).

### Flow Cytometry Analysis

Murine cells from *in vitro* experiments and those harvested from engrafted mice were fixed with 4% formaldehyde, washed, and permeabilized with methanol (on ice) for 1 h. After that, the cells were resuspended in PBS+0.1% Triton X-100+3% BSA and blocked with purified anti-mouse CD16/32 (10 μg/mL) (BioLegend) and 5% normal goat serum. The cell suspension was stained intracellularly with monoclonal antibody anti-human/mouse WASP (F8) (Santa Cruz Biotechnology) and purified mouse IgG2a isotype control (BioLegend) in PBS+0.1% Triton X-100+3% BSA buffer. After washing, a secondary antibody goat anti-mouse IgG2a-FITC conjugate (adsorbed against human Igs) (SouthernBiotech, Birmingham, AL, USA) was used. The antibodies used for surface staining were as follows: Brilliant Violet 421 anti-mouse/human CD45R/B220, Brilliant Violet 421 anti-mouse CD3, PE anti-mouse/human CD11b, Brilliant Violet 421 anti-mouse Ly6G/Ly6C (Gr1) (all from BioLegend), and APC mouse anti-mouse CD45.2 (BD Pharmingen). Samples were analyzed using a FACS LSR II flow cytometer (Becton Dickinson, NJ, USA) and the analysis software FlowJo.

For counting murine PLTs, blood was collected in tubes containing 20 μL of heparin and measured in the Sysmex XE-5000 automated hematology system (Sysmex, Japan).

Human primary cells were stained with anti-CD34-PE-Cy7, anti-CD41a-PE, and CD42b-APC (all from eBioscience) and cells lines with anti-CD41a-PE and CD42b-APC to evaluate megakaryocytic differentiation. Cells were incubated during 30 min at 4°C and washed with PBS at 300 × *g* for 5 min prior to acquisition. For intracellular WASP determination, 10^5^ cells were washed and fixed with 2% PFA during 20 min at RT. Permeabilization was performed with 0.2% saponin (Sigma-Aldrich) in PBS+3% BSA (Sigma-Aldrich) and blocking with FcR blocking reagent (Miltenyi Biotec) and 5% of normal goat serum (Abcam). Cells were then incubated 1 h on ice with anti-WASP (1:50, EP2541Y, Abcam) or rabbit IgG isotype control (Abcam). Secondary goat anti-rabbit IgG-FITC (AB6717, Abcam) was added at 1:1,000 during 40 min on ice. Acquisition was performed on a FACSCanto II (BD Biosciences) cytometer. Data were analyzed with FlowJo (Tree Star) and FACSDiva (BD Biosciences) software. Gates and analysis strategy for WASP and EGFP expression are shown in Figures S1, S3, S4, and S5A. Briefly, WASP expression is referred to as MeFI of the WASP^+^ population/MeFI of the isotype control or WASKO population, and fold expression uses as a control the expression of non-differentiated cells (CD41^−^CD42^−^ or CD34^+^, depending on the model):

$$Expression=\frac{WASP\;MeFI\;of\;WASP+cells\;in\;selected\;population}{WASP\;MeFI\;of\;IsC\;in\;total\;selected\;population\;}$$

$$Fold\;expression=\frac{Expression\;in\;selected\;population}{Expression\;in\;undifferentiated\;population\;}$$

### Statistical Analysis

Statistical comparisons were performed with GraphPad Prism software (GraphPad, San Diego, CA, USA). A non-parametric test (Mann-Whitney test), two-tailed p value (statistical significance was defined as a p value <0.05), two-way ANOVA, Bonferroni Post-Test, and an unpaired t test were used. All data are expressed as mean ± SEM.

## Author Contributions

P.M., M.T.-M., and A.S.-G.: experimental design, collection, and/or assembly of data, data analysis and interpretation, manuscript writing, and final approval of manuscript. G.S.: discussion and final approval of manuscript. A.G.: contributed reagents, discussion, and final approval of manuscript. A.J.T.: financial support, discussion, and final approval of manuscript. F.M.: conception and design, financial support, data analysis and interpretation, manuscript writing, and final approval of manuscript.

## Conflicts of Interest

The authors declare no competing interests.

## Supplemental Information

Document S1. Supplemental Materials and Methods and Figures S1–S11

Document S2. Article plus Supplemental Information
