# Supplementary material for: WAS Promoter-Driven Lentiviral Vectors Mimic Closely the Lopsided WASP Expression during Megakaryocytic Differentiation
Source: Mol Ther Methods Clin Dev. 2020 Sep 16;19:220–35. doi: 10.1016/j.omtm.2020.09.006 (PMC7558809; doi:10.1016/j.omtm.2020.09.006)
Supplement: Document S1. Supplemental Materials and Methods and Figures S1–S11 [file mmc1.pdf]

OMTM, Volume *19*

Supplemental Information

WAS Promoter-Driven Lentiviral Vectors Mimic Closely the Lopsided WASP Expression during Megakaryocytic Differentiation

Pilar Muñoz, María Tristán-Manzano, Almudena Sánchez-Gilabert, Giorgia Santilli, Anne Galy, Adrian J. Thrasher, and Francisco Martin

SUPPLEMENTAL MATERIAL & METHODS

Colony Formation Units (CFUs)

Gene-modified lin- cells were expanded for one day and 3,000-5,000 cells were plated in methylcellulose (MethoCult™ GF M3534, StemCell Technologies, Vancouver, Canada) to allow myeloid differentiation (in duplicates). Cells were incubated at 37°C, 5% CO_2 humidified atmosphere. Colonies were counted based on their morphological characteristics after 9 to 12 days and *WAS* mRNA expression levels analysed by RT-qPCR.

RT-qPCR for murine and human WAS expression in CFUs

For WAS expression, RNA was obtained from the transduced cells using the RNeasy Mini Kit (Qiagen) following manufacture´s indications. 5□l of each sample was amplified with iTaq™ Universal One-Step RT-qPCR kit (Biorad) in a reaction consisted of 10 min (50º C) 2 min (95º C) and 40 cycles at 15 sec (95º C) and 1 min (60º C). The murine *GAPDH* gene was used as control and relative expression was calculated using de ΔCT method [1]. To amplify the vector, we used sequences annealing the human and murine *WAS* described by Charrier et al [2]. The sequences of the primers are as follows:

hWAS-forward: 5´-TGATGCACGTGATGCAGAAGA-3´
hWAS-reverse: 5´-GGAGCACAGGGCAGCAAGT-3´
mWAS-forward: 5´-ACGGTCGACATTCAGAACCC-3´
mWAS-reverse: 5´-GGTGCTCCGATATCAGCTTTG-3´
mGAPDH-forward: 5´-TGTGTCCGTCGTGGATCTGA-3´
mGAPDH-reverse: 5´-CCTGCTTCACCACCTTCTTGA-3´

Myeloid in vitro differentiation

$1x10^{6}$ cells/ml were incubated during 7 days in RPMI supplemented with 20% FCS (all from Invitrogen), 1% penicillin/streptomycin, 20 ng/ml of mIL3 and 100 ng/ml of Granulocyte Macrophage Colony-Stimulating Factor (mGM-CSF) (all from Peprotech).

Hemograms

Blood from transplanted mice was collected, with heparin as anticoagulant, and the cells were counted with an XE-5000™ automated hematology system (Sysmex).

SUPPLEMENTAL FIGURES

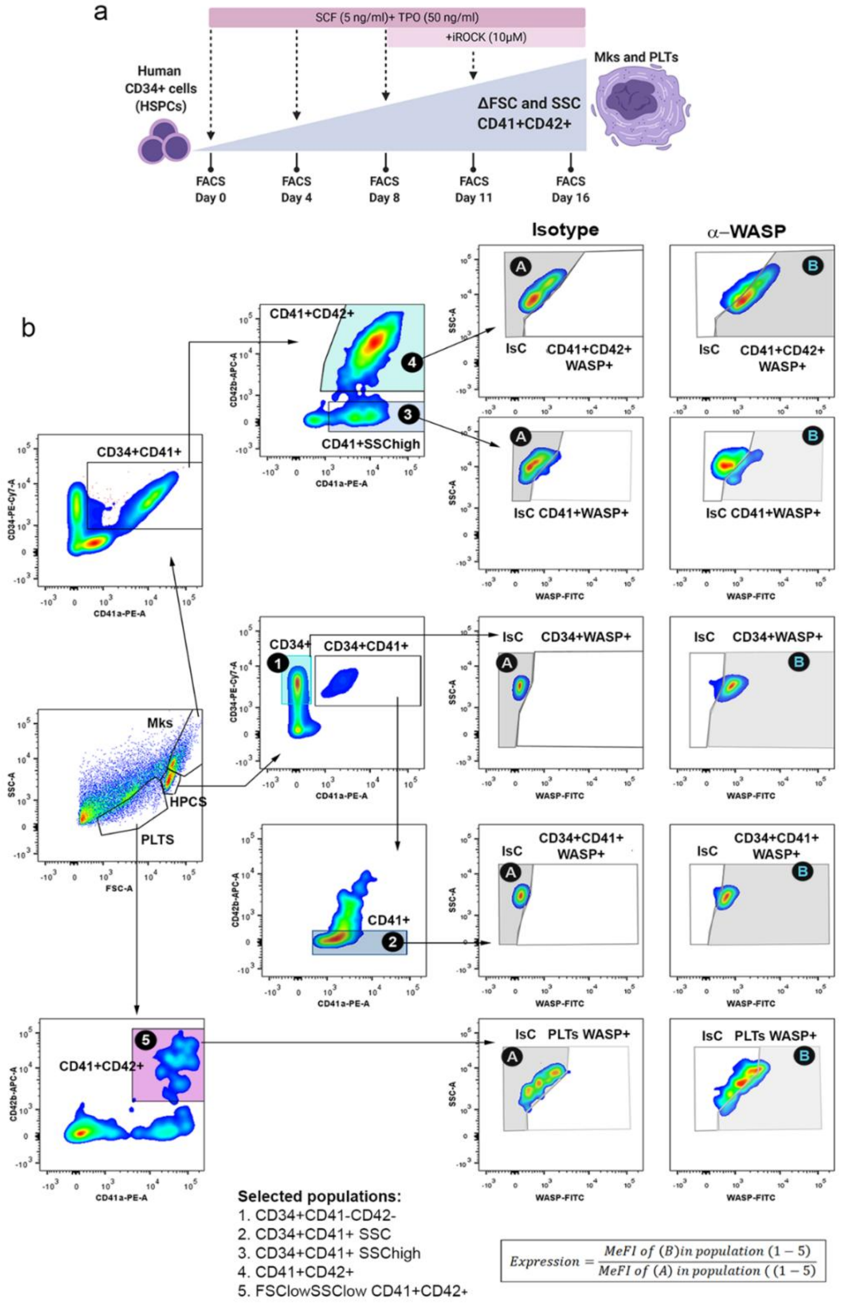

Figure S1. Megakaryocytic development from HSPCs and flow cytometry strategy for WASP analysis. a) HSPCs were cultured in StemSpan supplemented with TPO, SCF and iROCK (from day 8 of differentiation). b) Three gates according to FSC and SSC were established: PLTs ($FSC^{low}SSC^{low}$), HSPCs ($FSC^{mid}SSC^{low}$) and MKs ($FSC^{high}SSC^{high}$). Then, levels of CD34 and $CD41^{+}$ were determined in HSPCs and MKs regions. We selected a $CD34^{+}$ population that will correspond with the most undifferentiated HSPCs (Population 1). Next, we gated $CD34^{+}$ $CD41^{+}$ in the HSPCs region and selected $CD41^{+}SSC^{low}$ progenitor population (population 2). In the MKs region, we gated $CD34^{+}$ $CD41^{+}$ cells and differentiate $CD41^{+}SSC^{high}$ population (population 3) and mature MKs ($CD41^{+}$ $CD42^{+}$ cells)(population 4). Mature PLTs were defined as $CD41^{+}$ $CD42^{+}$ cells inside the PLTs region (population 5). WASP expression was analysed in those defined populations and referred as the MeFI of positive WASP+ cells (Region B)/ MeFI of Isotype Control (Region A, grey color) or WASKO population.

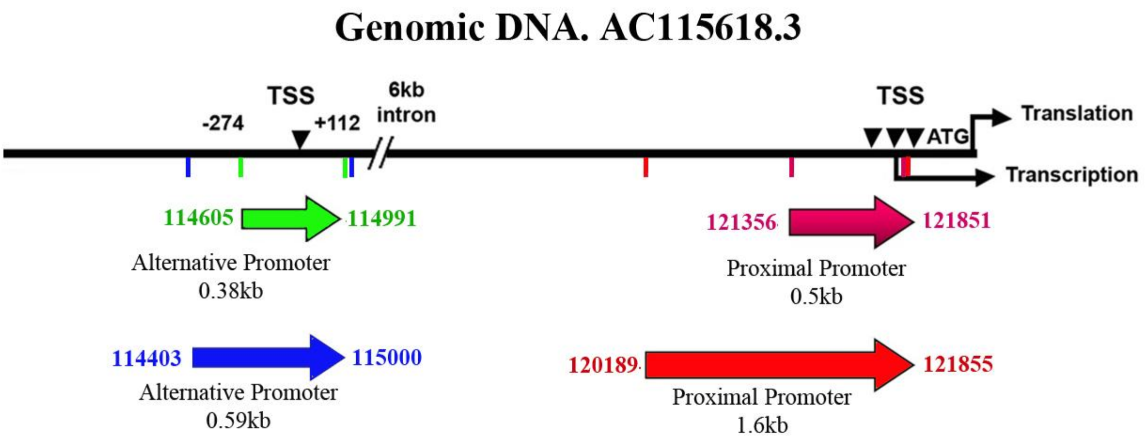

Figure S2. Scheme showing the different promoter regions of the WASP promoter used for the construction of the different lentiviral vectors. Genebank accession number AC115618.3 is used for reference. The WE and the WW vectors contains a WAS proximal promoter of 0.5kb (pink) (Seq 121356-121851). The WW1.6 clinical vector contains the full fragment of the proximal promoter (Red) (Seq 120189-121855). The AWE and AWW vectors contain a chimeric promoter harbouring a 0.38kb fragment of the alternative promoter (green) (AC115618.3 Seq 114605-114991) and the 0.5kb fragment of the proximal promoter (pink) (AC115618.3 Seq 121356-121851) linked by a 17nt fragment containing the EcoRI site. Finally, the cAWE and cAWW vectors contain a chimeric promoter harbouring a 0.59kb fragment of the alternative promoter (Blue)(AC115618.3 Seq 114403-115000) and the 0.5kb fragment of the proximal promoter (pink) (AC115618.3 Seq 121356-121851) linked by a 17nt fragment containing the EcoRI site.

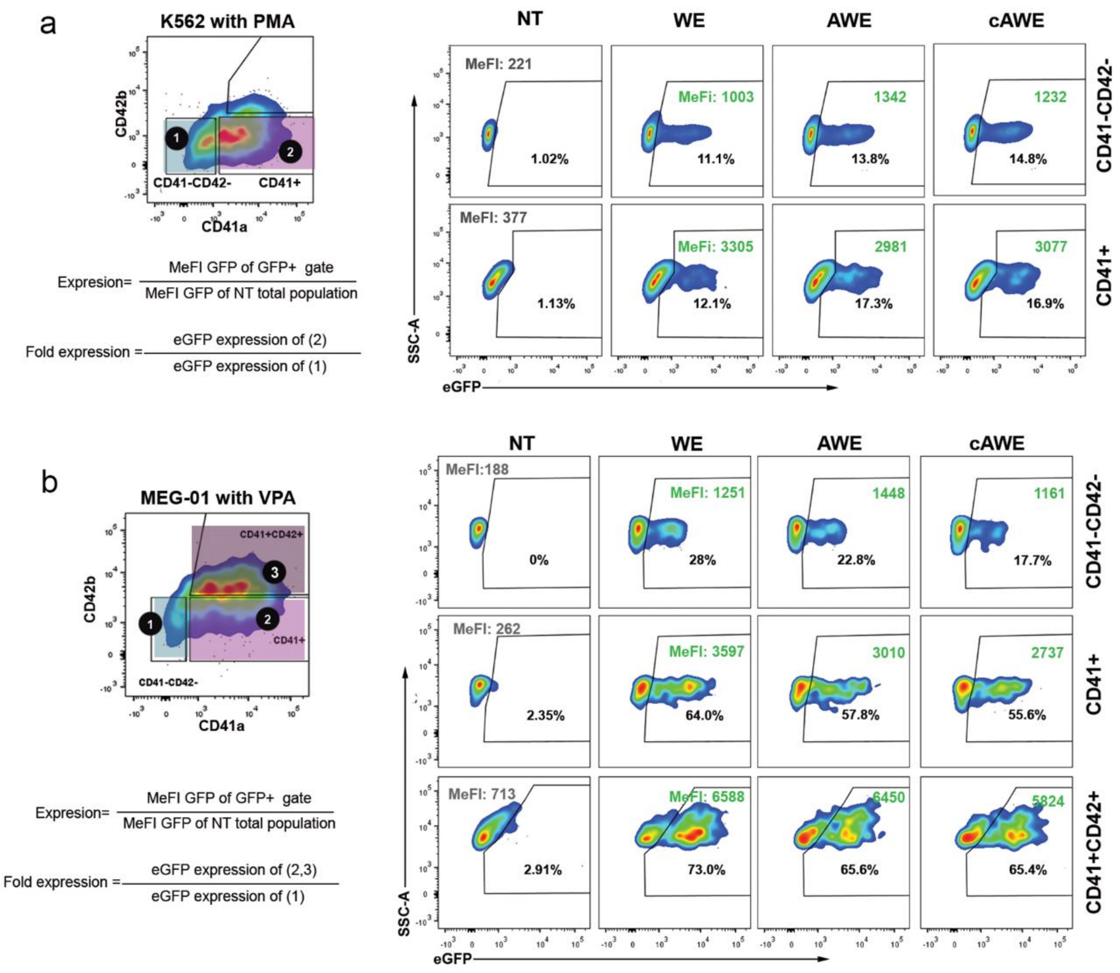

Figure S3. Gating strategy for eGFP-LVs analysis in K562 and MEG-01 cellular models. A) *Left panel*. Two populations of K562 cells according were selected in the presence of PMA: CD41-CD42- (population 1) and CD41+ (population 2). *Expression* was calculated as the MeFI of GFP in the GFP+ gate (transduced cells) and divided by expression GFP MeFI of non-transduced cells (NT) in CD41-CD42- or CD41+ cells. *Fold expression* is the ratio of expression in the differentiated population (population 2, CD41+) relative to those expression of undifferentiated cells (population 1, CD41- CD42- cells). *Right panel*. Representative dot-plot of NT (non-transduced) K562 and WE-, AWE-, cAWE transduced K562 cells. Background MeFI in non-transduced cells is indicated in grey and MeFI of transduced population (eGFP+ cells) in green for every LV and population. B) *Left panel*. Three populations of MEG-01 cells in the presence of VPAwere established: CD41-CD42- (population 1), CD41+ (population 2) and CD41+CD42+ (population 3). *Expression* was calculated as the MeFI of GFP in the GFP+ gate (transduced cells) and divided by GFPMeFI of non-transduced cells (NT) in every defined population (1-3).*Fold expression* is the ratio of expression in differentiated populations (populations 2 and 3) relative to those expression of undifferentiated cells (population 1, CD41- CD42- cells).*Right panel*.Representative dot-plots of NT (non-transduced) MEG-01 and WE-, AWE-, cAWE transduced MEG-01 cells. Background MeFI in non-transduced cells is indicated in grey and MeFI of transduced population (eGFP+ cells) in green for every LV and population.

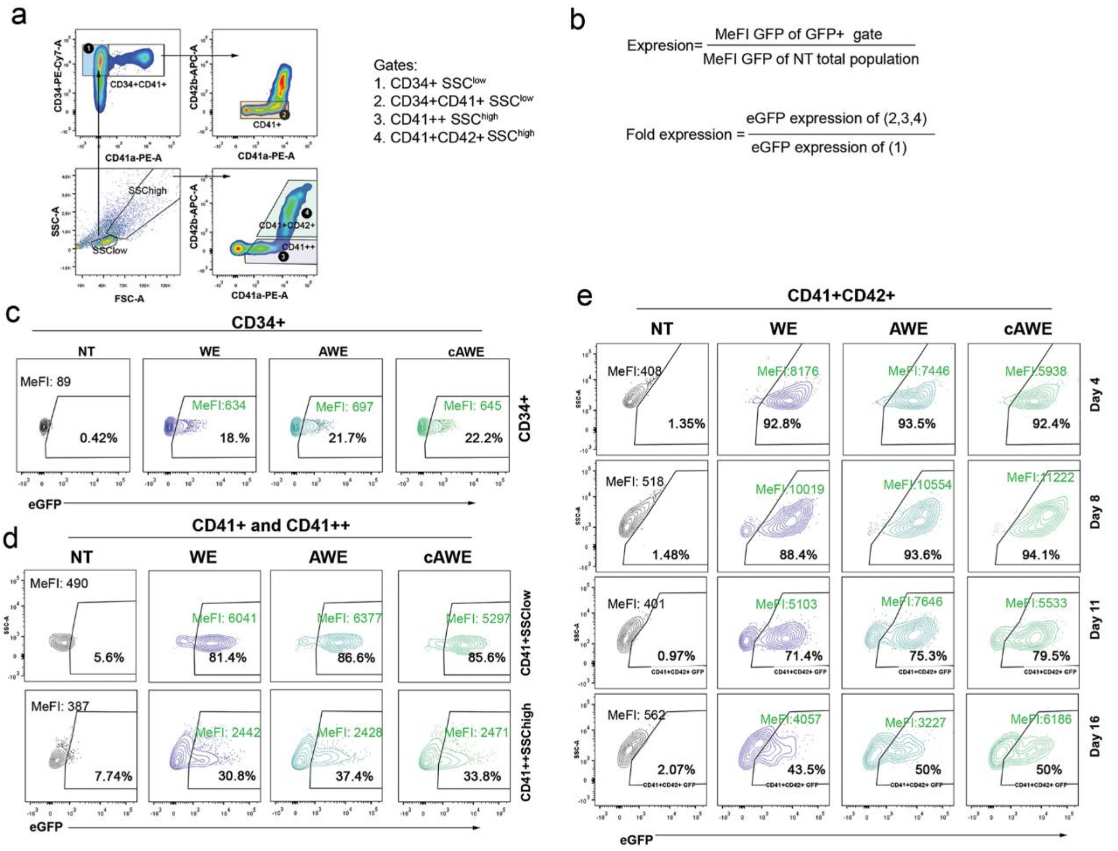

Figure S4. Gating strategy for eGFP-LVs analysis in HPSCs and megakaryocytic populations. a) Four populations according to FSC, SSC and CD34, CD41a and CD42b expression markers were defined: SSC^{low} CD34+ (population 1), CD34+CD41+ (population 2) and SSC^{high} CD41+ (population 3) and CD41+CD42+ (population 4) gates. b) Expression and fold expression used formulas. Representative dot-plots of eGFP expression given by WE, AWE and cAWE LVs in c) CD34+ HSPCs (population 1), d) in progenitors CD41+SSC^{low} (population 2) and CD41++SSC^{high} (population 3) and e) and in MKs (population 4). Percentage and MeFI are indicated. Background MeFI in non-transduced cells is indicated in grey and MeFI of transduced population (eGFP+ cells) in green for every LV and population.

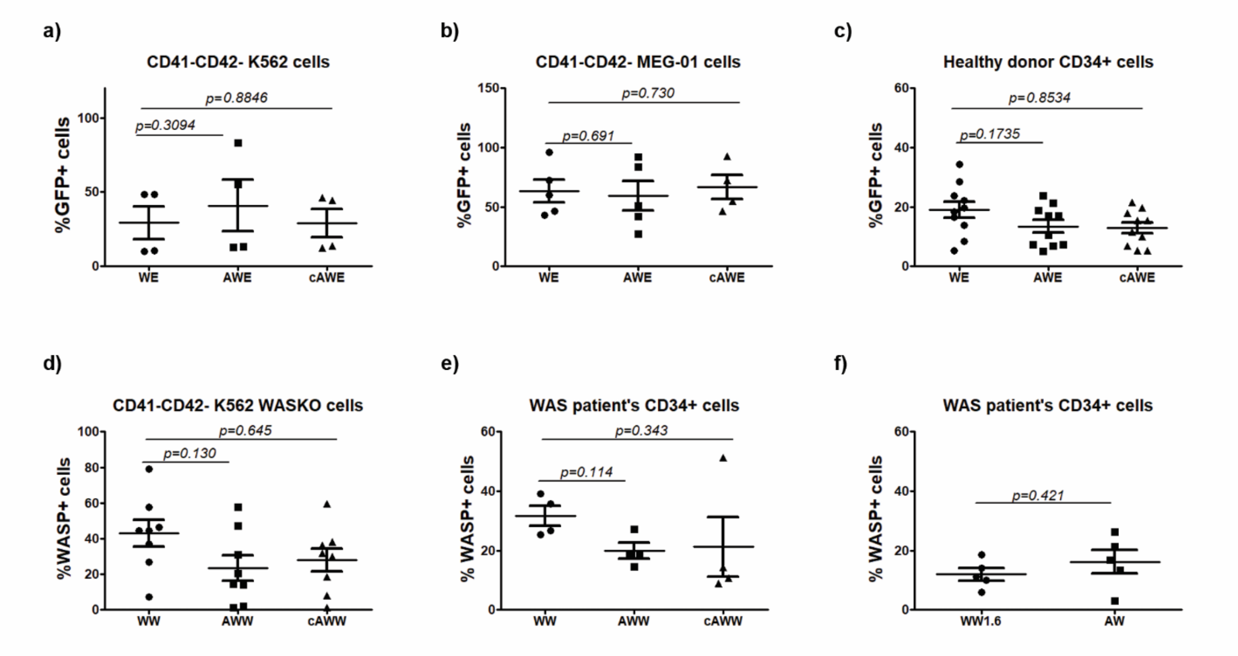

Figure S5. Transduction efficacy of eGFP and WAS-driven LVs in undifferentiated K562, MEG-01 and HSPCs. a) Percentage of eGFP+ cells in CD41-CD42- K562 cells transduced with WE, AWE and cAWE at MOI=1.(N=4) b) Percentage of eGFP+ cells in CD41-CD42- MEG-01 cells transduced with WE, AWE and cAWE at MOI=1. (N=4)c) Percentage of eGFP+ cells in CD34+CD41-CD42- human HSPCs cells transduced with WE, AWE and cAWE at MOI=50 (N=10). d) Percentage of WASP+ cells in CD41-CD42- K562-WASKO cells transduced with WW, AWW and cAWW at MOI=1 (N=7). e) Percentage of WASP+ cells in WAS patient’s CD34+ CD41-CD42- HSPCs transduced with WW, AWW and cAWW at MOI=50 (N=4). f) Percentage of WASP+ cells in WAS patient’s CD34+ CD41-CD42- HSPCs transduced with WW1.6 and AW at MOI=50(N=5).

a)

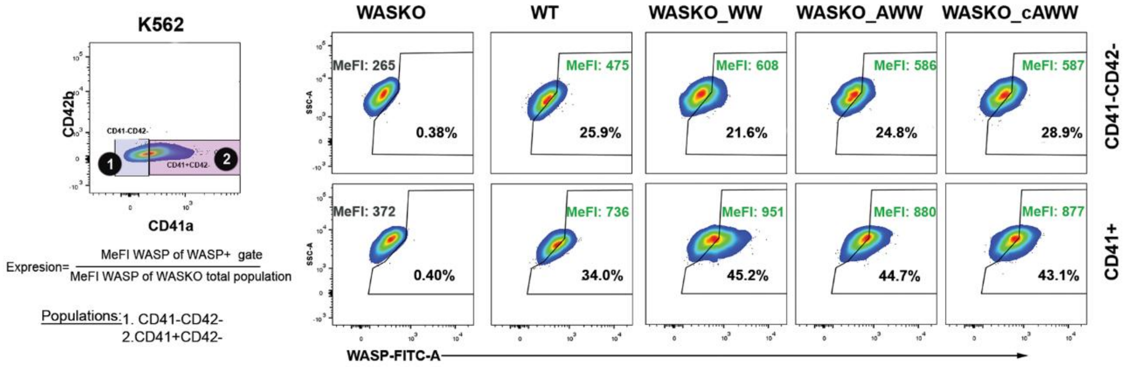

Figure S6. WAS-LVs analysis in K562-KO cells during megakaryocytic differentiation. a)*Left panel.* Two populations were defined in K562-WASKO cells treated with PMA: CD41-CD42- (**population 1**) and CD41+ (**population 2**). Expression was calculated as the MeFI of WASP in the WASP+ gate (transduced cells) divided by the background MeFI of WASP staining in WASKO- negative cells. *Right panel.* Representative dot-plots of K562 WASKO cells, WT and WASKO transduced with WW, AWW and cAWW LVs in both defined populations. Both percentage and MeFI were shown. MeFI in grey indicates WASP background in WASKO cells. MeFI in green is referred to WASP+ gate. b) Percentage of WASP+ cells in CD41-CD42- and CD41+ cells in K562 WT and WASKO-transduced with the second-generation LVs. Non-parametric Mann-Whitney T-Test, two tails (N=4).

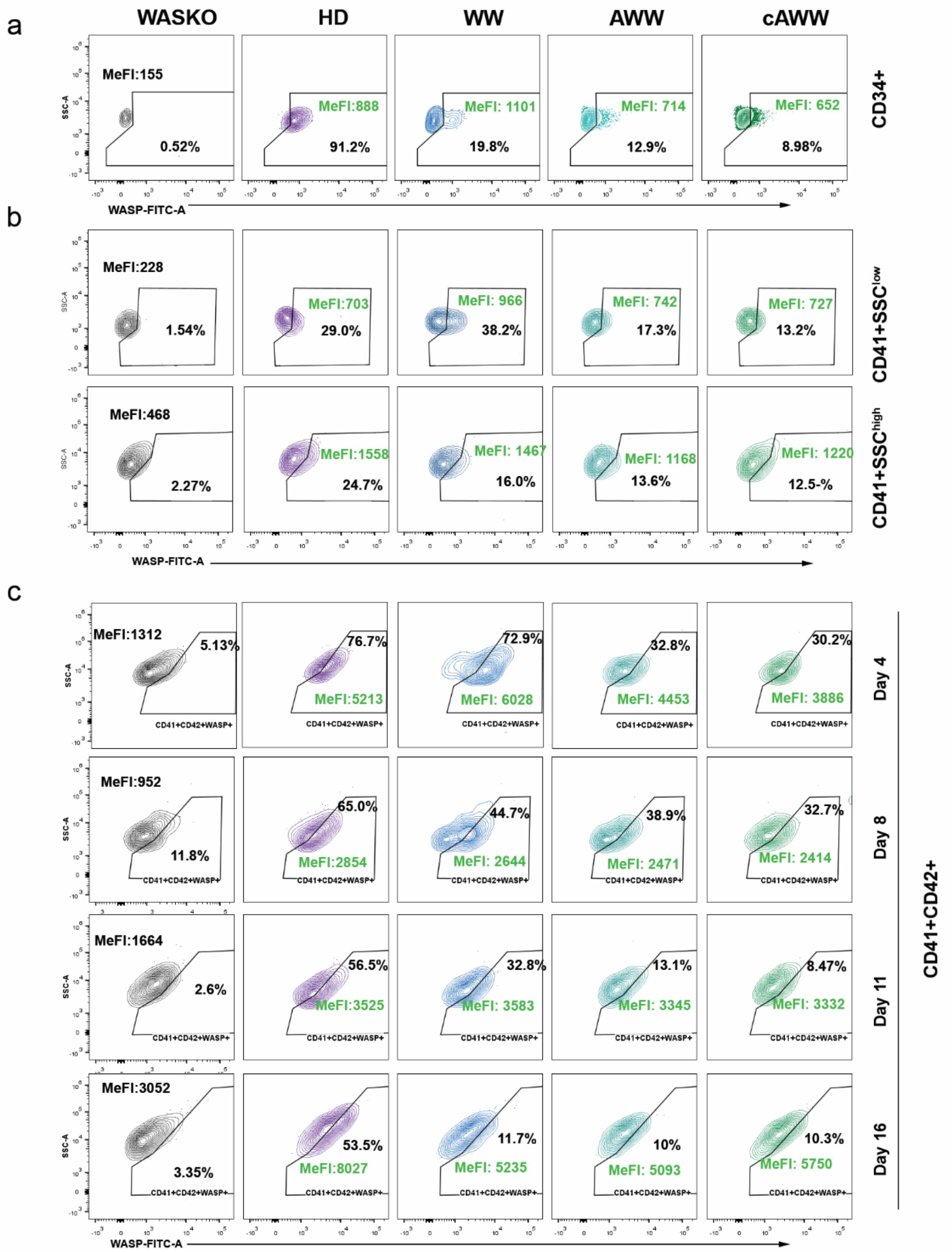

Figure S7. Representative dot-plots showing the behavior of WAS- second generation LVs in WAS patient's HSPCs during megakaryocytic differentiation. Representative dot-plots of WASP expression given by the second-generation LVs WW, AWW and cAWW LVs in a) CD34+ HSPCs (population 1), b) in progenitors CD41+SSC^{low} (population 2) and CD41++SSC^{high} (population 3) and c) and in MKs (population 4). Percentage and MeFI are indicated. Background MeFI in WASKO cells is indicated in grey and MeFI of transduced population (WASP+ cells) in green for every LV and population.

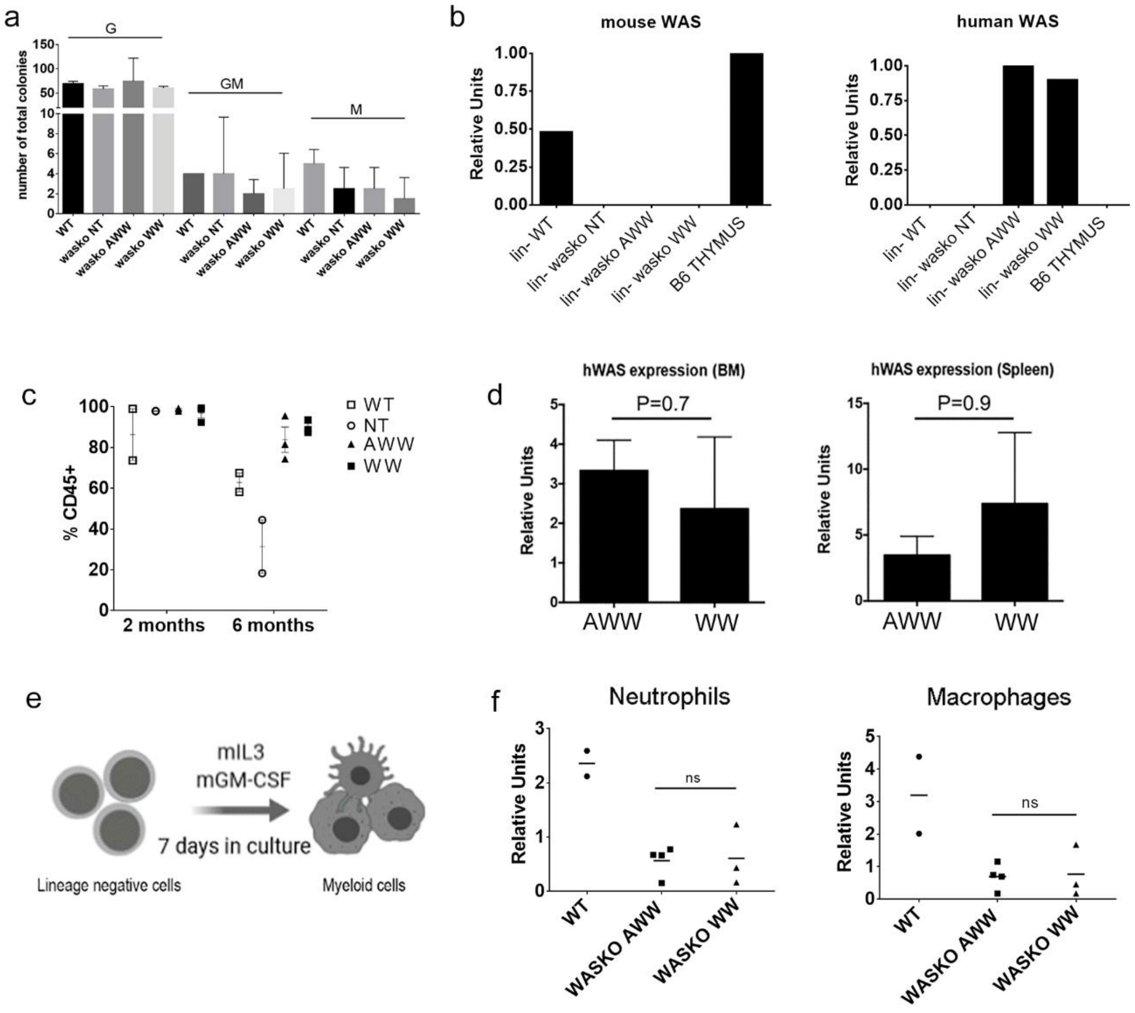

Figure S8. mHSPCs transduced with AWW or WW LVs give arise methylcellulose myeloid derived colonies expressing hWASP are able to engraft in WASKO mice (long-term experiment). a) AWW-transduced lineage negative (lin-) cells maintain the colony forming potential in methylcellulose and express *WAS* gene. Lin- cells were isolated from bone marrow of *WAS*$^{-/-}$ mice by Miltenyi Microbeads and transduced with the LVs AWW and WW at MOI=100 achieving 9.8vcn/c and 8.2vcn/c respectively. As controls we used non-transduced lin- cells from *WAS*$^{-/-}$ (WASKO NT) and lin- cells from wild-type mice (WT). The different cells were expanded for one day and 3.000 cells (duplicates) were placed onto methylcellulose to allow myeloid differentiation. b) Murine (left) and human *WAS* (right) expression levels in myeloid colonies (mRNA). c) AWW and WW transplanted mice showed engraftment (mCD45$^+$) two- and six-months post-transplant. The engraftment did not decrease dramatically in AWW or WW-WASKO transplanted mice compared with non-transduced-WASKO transplanted mice (peripheral blood) and they have also a with similar engraftment (% CD45$^+$ cells) compared with the wild-type group (after 6 months). d) Efficient expression of human WASP in bone marrow (left) and spleen (right) of WASKO mice engrafted with AWW-transduced mHSPCs. Lin- cells were isolated, transduced with the AWW and WW at MOI 100 and inoculated in lethally irradiated WASKO mice. Human *WAS* expression was analysed 7 months post-transplant. Human *WAS* was determined by RT-qPCR using primers specific for the vector mRNA encoding human *WAS*. Data represent mean ± SEM. 3 mice per group. AWW-mHSPCs (bone marrow): 0.89±1.16copies/cell. WW0.5-mHSPCs (bone marrow): 2.21±2.96 copies/cell. e) General protocol for myeloid differentiation of lineage negative cells (lin-) towards myeloid lineage with media supplemented with mIL3 (20 ng/ml) and mGM-CSF (100 ng/ml). f) hWASP+ expression in neutrophils and macrophages (CD11b$^+$ F4/80$^+$) after differentiation *in vitro* of AWW and WW-WASKO lin- cells (2 differentiations *in vitro*). The relative units were calculated by dividing the median of fluorescence (MeF) of AWW and WW-transduced cells by MeF (FITC) of non-transduced cells (NT) and corrected by their number of copies/cell. Data represent mean ± SEM.

a

c/c	Spleen	Bone Marrow	Blood
AWW 1L	2±1	0.3±0.1	2±0.5
AWW 1R	0.2±0.1	0.2±0.1	1.4±0.7
AWW -	1.4±0.6	2.2±1.1	1.5±0.8
WW 1L	0.2±0.2	0.1±0	0.9±0.4
WW 1R	15.1±6.1	5.6±1	8.6±1.4
WW -	4.7±3.4	0.9±0.1	6.7±0.4

b

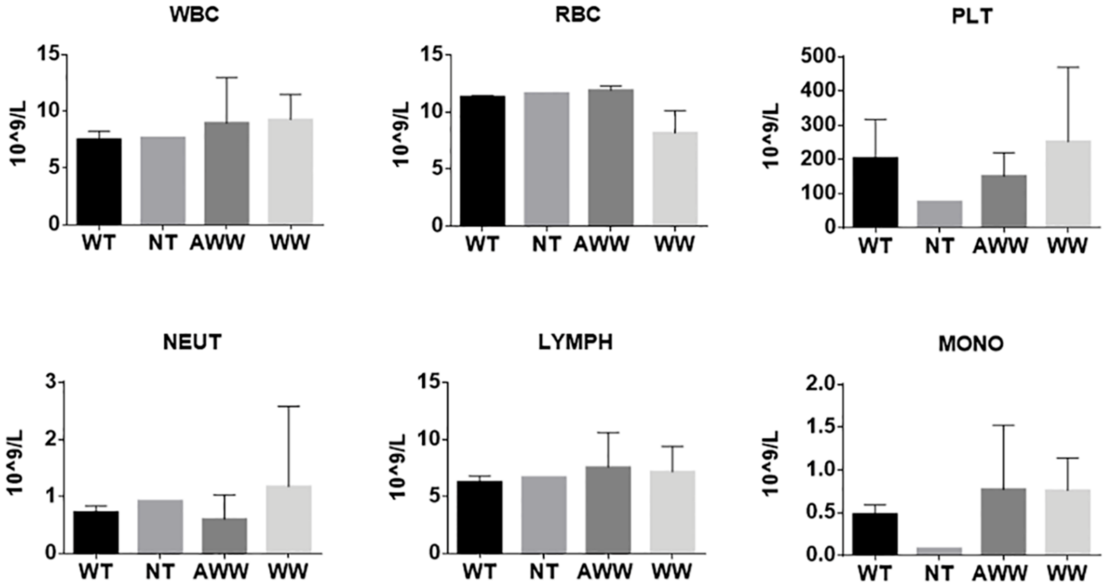

Figure S9. Copies/cell in organs and hemograms of AWW- and WW- transplanted mice. a) Copies/cells of the LVs were analysed in spleen, bone marrow and blood of long-term transplanted mice, AWW (n=3) and WW (n=3), 7 months. b) Lin- cells from WASKO mice were transduced with the AWW and WW LVs (MOI=100) overnight (see M&M). 500.000 cells were transplanted per mouse and blood samples were analyzed 3 months after transplant. Cells were counted with an XE-5000^{TM} automated hematology system (Sysmex).WBC= whole blood cells, RBC= red blood cells, PLT= platelets, NEUT= neutrophils, LYMPH= lymphocytes and MONO= monocytes. Mean ± SD.

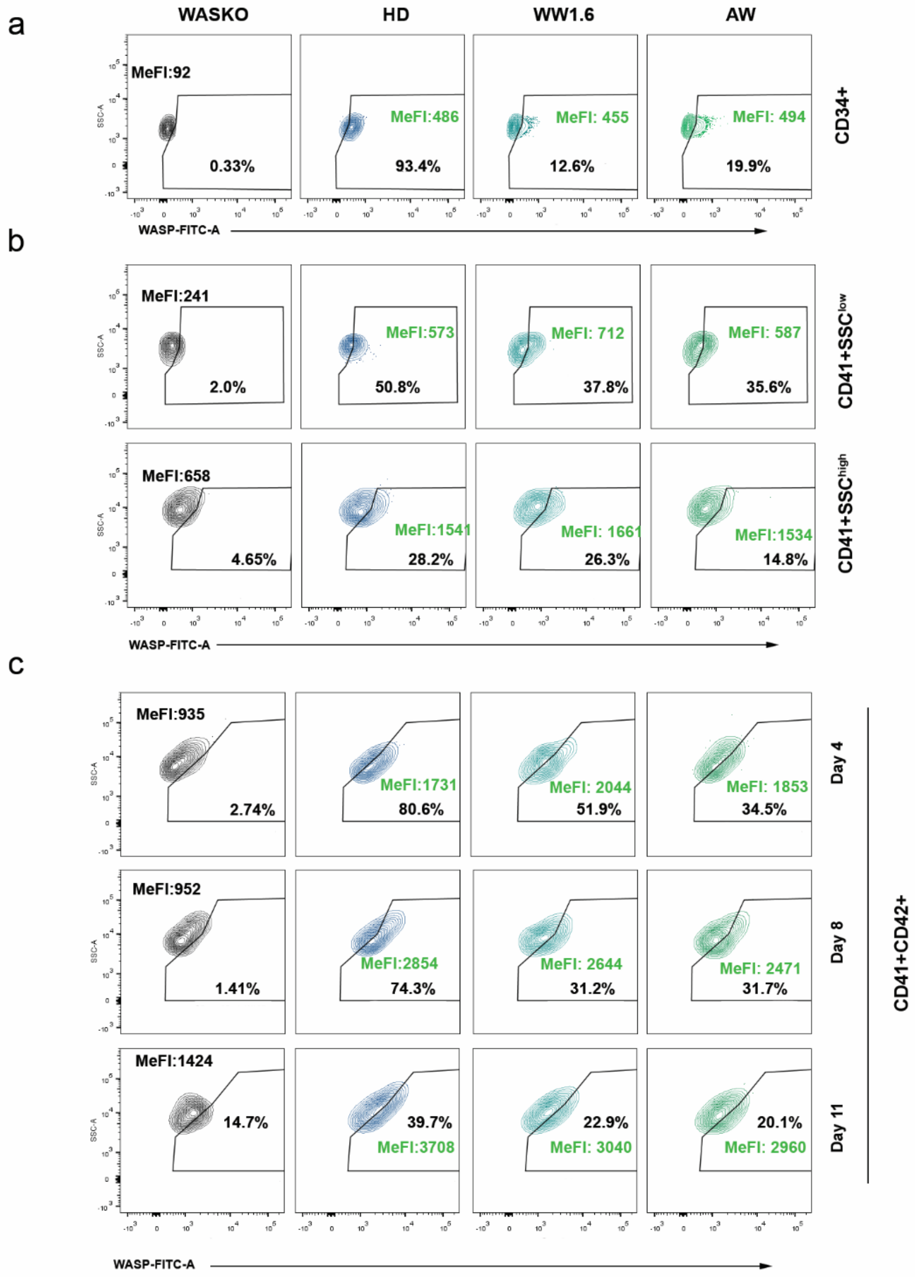

Figure S10. Representative dot-plots of WAS- clinical LVs behavior during megakaryocytic differentiation from HSPCs of WAS patient's cells. Representative dot-plots of WASP expression given by the second-generation LVs WW, AWW and cAWW LVs in a) CD34+ HSPCs (population 1), b) in progenitors CD41+SSC^{low} (population 2) and CD41++SSC^{high} (population 3) and c) and in MKs (population 4). Percentage and MeFI are indicated. Background MeFI in WASKO cells is indicated in grey and MeFI of transduced population (WASP+ cells) in green for every LV and population.

a)

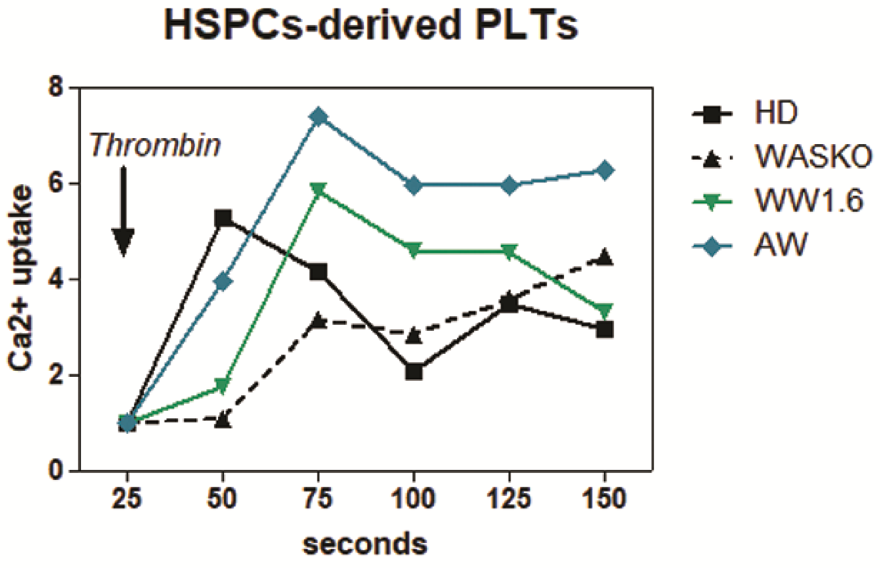

Figure S11. Calcium uptake after thrombin stimulation in platelets derived of WASKO-transduced HSPCs with clinical vectors. PLTs obtained *in vitro* from the megakaryocytic differentiation of patient's HSPC cells at day 11 were stained with CD42b and calcium-eFluor514 as described in M&M. Basal levels of calcium were acquired during 30 seconds on the FACsCanto II flow cytometer. Thrombin (2 units/ml) were added and acquired immediately during 120 seconds. (N=1). HD, healthy donor. WAS, WAS patient

REFERENCES

1. Livak, K.J. & Schmittgen, T.D. (2001) Analysis of relative gene expression data using real-time quantitative PCR and the 2(-Delta Delta C(T)) Method. *Methods* **25**, 402-408
2. Charrier, S., Stockholm, D., Seye, K., Opolon, P., Taveau, M., Gross, D.A. et al. (2005) A lentiviral vector encoding the human Wiskott-Aldrich syndrome protein corrects immune and cytoskeletal defects in WASP knockout mice. *Gene Ther* **12**, 597-606.
